# A Dual-Plasmid CRISPR/Cas9-Based Genome Editing System for Efficient Gene Knockout and Large DNA Integration in *Cronobacter malonaticus*

**DOI:** 10.3390/pathogens15090990

**Published:** 2026-09-17

**Authors:** Shuwei Yan, Jing Zhang, Yufei Han, Jia Jia, Ge Yun, Dan Liu, Yue Ma, Zhimeng Liu, Yuanyuan Wang, Haifeng Wang

**Affiliations:** 1 Department of Laboratory, Xi’an No.3 Hospital, the Affiliated Hospital of Northwest University, Xi’an 710018, China; yanshuwei1993@163.com; 2 Key Laboratory of Resource Biology and Biotechnology in Western China, Ministry of Education, Faculty of Life Science and Medicine, Northwest University, Xi’an 710069, China; 3 School of Medicine, Northwest University, Xi’an 710069, China; 4 College of Medical Technology, Shaanxi University of Chinese Medicine, Xi Xian New Area, Xi’an 712046, China

**Keywords:** *Cronobacter*, CRISPR-Cas9, genome editing, homologous recombination, large-fragment integration

## Abstract

Species of *Cronobacter* are emerging foodborne pathogens that pose a significant threat to neonates. Functional genomic studies in *Cronobacter* have been hindered by the lack of efficient genetic manipulation tools. Here, we established a CRISPR/Cas9-based genome editing platform for *Cronobacter*. We developed a dual-plasmid platform, pAmpCRISPR/pCasCm, by integrating CRISPR/Cas9 with the λ-Red recombination machinery. This platform enables scarless genome editing in *Cronobacter malonaticus* and proved effective for gene knockout and large-fragment integration under the tested conditions. Furthermore, we validated this platform in another clinically relevant species, *Cronobacter sakazakii*. The development of this genome editing toolkit provides a useful approach for fundamental research into *Cronobacter* pathogenesis, such as bacterial physiology studies, drug target exploration, and metabolic engineering.

## 1. Introduction

*Cronobacter* spp. are Gram-negative, motile, non-spore-forming, rod-shaped bacteria belonging to the family Enterobacteriaceae. As a foodborne pathogen, *Cronobacter* is widely present in the intestinal tracts of both humans and animals [1,2,3]. *Cronobacter* can infect immunocompromised adults (mainly the elderly) and infants [4]. Among them, infants are at high risk of *Cronobacter* infection, mainly associated with powdered infant formula (PIF) [5], leading to severe clinical outcomes such as necrotizing enterocolitis (NEC), sepsis, and meningitis. Notably, the case fatality rate of invasive *Cronobacter* infections in neonates ranges from 40% to 80% [6,7]. Currently, significant research efforts are focused on elucidating the molecular basis of infection mechanisms and developing novel therapeutic strategies against *Cronobacter* infections. Consequently, biocontrol methods targeting high-risk food products, such as powdered infant formula (PIF), can be implemented to mitigate *Cronobacter*-related infections in vulnerable populations [8].

Genome editing technology is a powerful tool that enables precise modifications of specific genomic targets within living organisms. By introducing site-specific DNA double-strand breaks (DSBs) and harnessing endogenous repair mechanisms, it achieves efficient and accurate genome manipulation—including insertions, deletions, or replacements—thereby altering genetic information and phenotypic traits at the molecular level [9,10]. To date, genome editing technologies have evolved through three generations. The first generation, zinc finger nucleases (ZFNs), relies on zinc finger proteins to recognize specific DNA sequences, with FokI nuclease mediating DNA cleavage [11]. However, ZFNs are limited by high costs and restricted target site flexibility [12]. The second generation, transcription activator-like effector nucleases (TALENs), utilizes TALE proteins fused to FokI for gene editing [13]. Compared to ZFN technology, this technique is easier to use but has a complex construction process [14]. The third and most advanced generation is the CRISPR/Cas system. Originally functioning as an adaptive immune system in bacteria and archaea to defend against invasive genetic elements such as phages and plasmids [15], CRISPR/Cas9 has been repurposed for genome editing [16]. Its core component, the Cas9 protein, is guided by a single-guide RNA (sgRNA) to precisely bind and cleave target DNA sequences [17]. Renowned for its simplicity and efficiency, CRISPR/Cas9 has become the most widely adopted genome editing tool [18].

Recent advancements in CRISPR/Cas technology have driven its rapid application across diverse organisms. For example, Jiang et al. (2015) developed a CRISPR/Cas9-based multiplex genome editing platform for *Escherichia coli* [19,20], while Chen et al. established high-efficiency CRISPR/Cas9 systems for *Staphylococcus aureus* and *Pseudomonas aeruginosa* [21,22]. The workflow involves designing a 20-nucleotide targeting sequence within the crRNA, which hybridizes with tracrRNA to form the sgRNA. The sgRNA-Cas9 complex is then delivered into cells, where it identifies the target DNA via complementary base pairing and recognition of a protospacer adjacent motif (PAM, e.g., 5′-NGG-3′). Cas9 subsequently induces DSBs, enabling precise genomic edits through homology-directed repair (HDR) using exogenous donor templates [23,24,25].

The development of efficient and rapid genome editing tools is critical to advancing research and applications involving *Cronobacter* spp. To the best of our knowledge, no studies have reported the implementation of CRISPR-Cas systems for genome editing in *Cronobacter*. In this study, we established a CRISPR-Cas9-based genome editing platform for *Cronobacter* by optimizing sgRNA design and Cas9 expression regulation. By integrating the targeted DNA cleavage capability of CRISPR-Cas9 with the λ-Red recombination system, we significantly enhanced the editing efficiency of the *C. malonaticus* (*Cronobacter malonaticus*) and *Cronobacter sakazakii* (*C. sakazakii*) genomes, achieving precise genetic modifications such as gene knockouts and large DNA fragment integrations. This provides important technical support for genomic research, investigations into virulence mechanisms, and metabolic pathway engineering in *Cronobacter*.

## 2. Materials and Methods

### 2.1. Bacterial Strains, Plasmids, and Growth Conditions

All bacterial strains, plasmids, and primers used in this study are listed in Tables S1 and S2. All *C. malonaticus* and *C. sakazakii* strains were cultured in Luria–Bertani (LB) broth at 37 °C or 30 °C as required. When necessary, media and agar plates were supplemented with carbenicillin (Sangon, Shanghai, China) (100 μg/mL) or tetracycline (Sangon, Shanghai, China) (10 μg/mL).

### 2.2. Plasmid Construction

The pAmpCRISPR plasmid was constructed as follows: fragments flanking the two Esp3I (NEB, MA) sites were amplified from pUCP20, and the *oriT* gene was amplified from pEX18Tc. These fragments were assembled by seamless cloning to generate the intermediate vector pUCP20T. The spacer-containing sgRNA cassette was amplified from pCasSA and fused with the tac promoter by overlap PCR. The *sacB* gene was amplified from pEX18Tc, and a *lac* promoter-deleted backbone was amplified from pUCP20T. All fragments were finally assembled by seamless cloning to generate the pAmpCRISPR plasmid (Data S1).

The pCasCm plasmid was constructed as follows: using the pKD46 plasmid as a template, a linearized vector backbone lacking the ampicillin resistance gene (AmpR) was amplified. Meanwhile, the tetracycline resistance gene (TcR) was amplified from pEX18Tc, while the Cas9 gene was amplified from pCasSA. These fragments were assembled by seamless cloning to generate the pCasCm plasmid (Data S2).

### 2.3. Motility Test

The motility assay for *C. malonaticus* was performed according to the method previously described by Li et al. [26] with minor modifications. Specifically, overnight bacterial cultures were spotted onto LB semi-solid agar plates containing 0.3% agar and incubated at 30 °C until visible motility halos formed.

### 2.4. Preparation of Electrocompetent Cells

Electrocompetent cells were prepared as previously described with minor modifications [27]. Overnight bacterial cultures were chilled on ice prior to centrifugation. The cells were then collected by centrifugation at 8000 rpm for 5 min at 4 °C. Subsequently, the cells were washed three times with ice-cold 0.3 mol/L sucrose solution under the same centrifugation conditions (8000 rpm, 4 °C, 5 min each). Finally, the cells were resuspended in 500 μL of 0.3 mol/L sucrose solution and aliquoted into 100 μL portions for electroporation experiments.

Electroporation efficiency and gene editing efficiency of the pAmpCRISPR plasmid were calculated as follows: electroporation efficiency (CFU/μg DNA) = number of colonies obtained/amount of plasmid DNA used. The electroporation experiment was performed as a single independent trial, and the editing efficiency was calculated as the number of PCR-verified positive clones divided by the total number of clones analyzed. For each electroporation, colonies were selected for PCR verification based on the following criteria: if more than 10 colonies were obtained, 10 were randomly selected; if fewer than 10 colonies were obtained, all were selected.

Preparation of electrocompetent cells harboring the pCasCm plasmid (*C.m*/pCasCm). Overnight cultures grown at 30 °C were inoculated into fresh 50 mL LB medium at a ratio of 1:100, supplemented with the appropriate antibiotics, and incubated at 30 °C with shaking at 180 rpm for 1 h. Subsequently, 500 μL of 20% L-arabinose was added to induce gene expression, and cultivation was continued until cells reached the exponential growth phase. The subsequent preparation steps were identical to those used for wild-type electrocompetent cells, except that the electrocompetent cells were used immediately after preparation.

### 2.5. Integration of the Lux Fragment

The *luxC*, *luxD*, *luxA*, *luxB*, *luxE* gene cluster (*luxCDABE*) was first amplified by PCR. Subsequently, the pAmpCRISPR-*fliC*-sgRNA-repair plasmid containing 1000 bp homology arms on each side was digested with XhoI (NEB, MA) between the upstream and downstream homology arms. The *luxCDABE* fragment was then inserted between the homology arms by Gibson assembly to generate the recombinant plasmid pAmpCRISPR-sgRNA*^fliC^*-repair*^luxCDABE^*. The recombinant plasmid was electroporated into the *C.m*/pCasCm strain. Positive clones were initially screened by colony PCR using primers targeting the insertion site. Functional validation was performed by assessing bioluminescence generated by the integrated *luxCDABE* operon. Ultimately, recombinant *C. malonaticus* strains with stable chromosomal integration of the *luxCDABE* gene cluster were selected based on stable luminescent phenotypes.

The complete sequences of the plasmids constructed in this study, as well as the detailed gene knockout procedures including sgRNA design, plasmid construction, and gene editing based on the dual-plasmid system, are provided in the Supplementary Materials.

## 3. Results

### 3.1. Construction of a CRISPR/Cas9-Based Dual-Plasmid Genome Editing System in C. malonaticus

**Figure 1:**
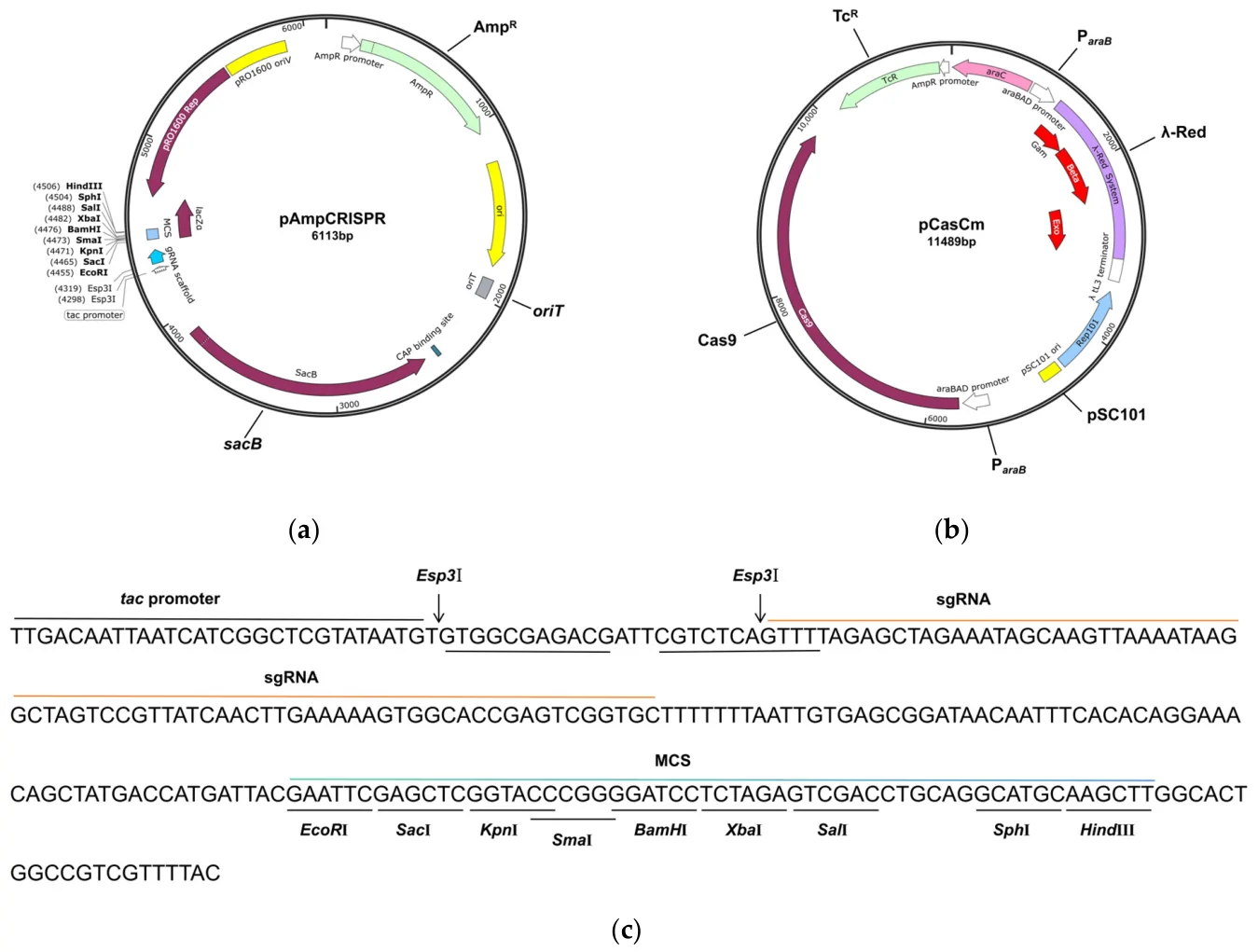
Plasmid map and cloning site distribution of the pAmpCRISPR/pCasCm system. (**a**) Plasmid map of pAmpCRISPR. *tac* promoter, the sgRNA expression promoter; MCS, multiple cloning site; Esp3I, the sgRNA sequence was cloned between two Esp3I restriction sites; Amp^R^, ampicillin resistance gene; *oriT*, origin of transfer; *sacB*, a sucrose counter-selectable marker. (**b**) Plasmid map of pCasCm. Expression of the λ-Red recombination system (Gam, Beta, and Exo) and Cas9 protein is driven by the L-arabinose-inducible *araB* promoter (P_*araB*_); pSC101, a temperature-sensitive replicon; Tc^R^, tetracycline resistance gene. (**c**) Cloning site sequence of the pAmpCRISPR plasmid.

To establish an efficient and streamlined genetic toolkit in *Cronobacter* species, we engineered a CRISPR/Cas9-based platform for genome editing in these bacteria. Based on the successful application of dual-plasmid CRISPR/Cas9 systems in *E. coli* and other bacteria [19,22], a dual-plasmid platform was designed and constructed in this study. This study successfully developed a dual-plasmid gene editing system (pAmpCRISPR/pCasCm) through integration of CRISPR/Cas9 and λ-Red recombination systems. The pAmpCRISPR plasmid primarily mediates sgRNA expression and contains a strong *tac* promoter-driven sgRNA cassette (Figure 1a) [28]. Two Esp3I restriction sites were engineered to enable rapid insertion of 20-nt targeting spacers via Golden Gate assembly, while the multiple cloning site supports homologous-arm cloning through Gibson assembly (Figure 1c). In addition, this plasmid incorporates a conjugative transfer *oriT* element [29] and the sucrose counter-selectable marker *sacB* gene, enabling efficient plasmid clearance after editing (Figure 1a). The pCasCm plasmid enables co-expression of the Cas9 nuclease and λ-Red recombination proteins (Exo, Gam, and Beta) [30], with expression precisely regulated by the L-arabinose-inducible P*_araB_* promoter [31]. Additionally, it employs a temperature-sensitive pSC101 replicon [32], which synergizes with the *sacB* selection mechanism in pAmpCRISPR to achieve dual-plasmid elimination (Figure 1b). This integrated design significantly improves the simplicity and efficiency of the gene editing system.

### 3.2. The Editing Efficiency of the pAmpCRISPR/pCasCm Dual-Plasmid System

**Figure 2:**
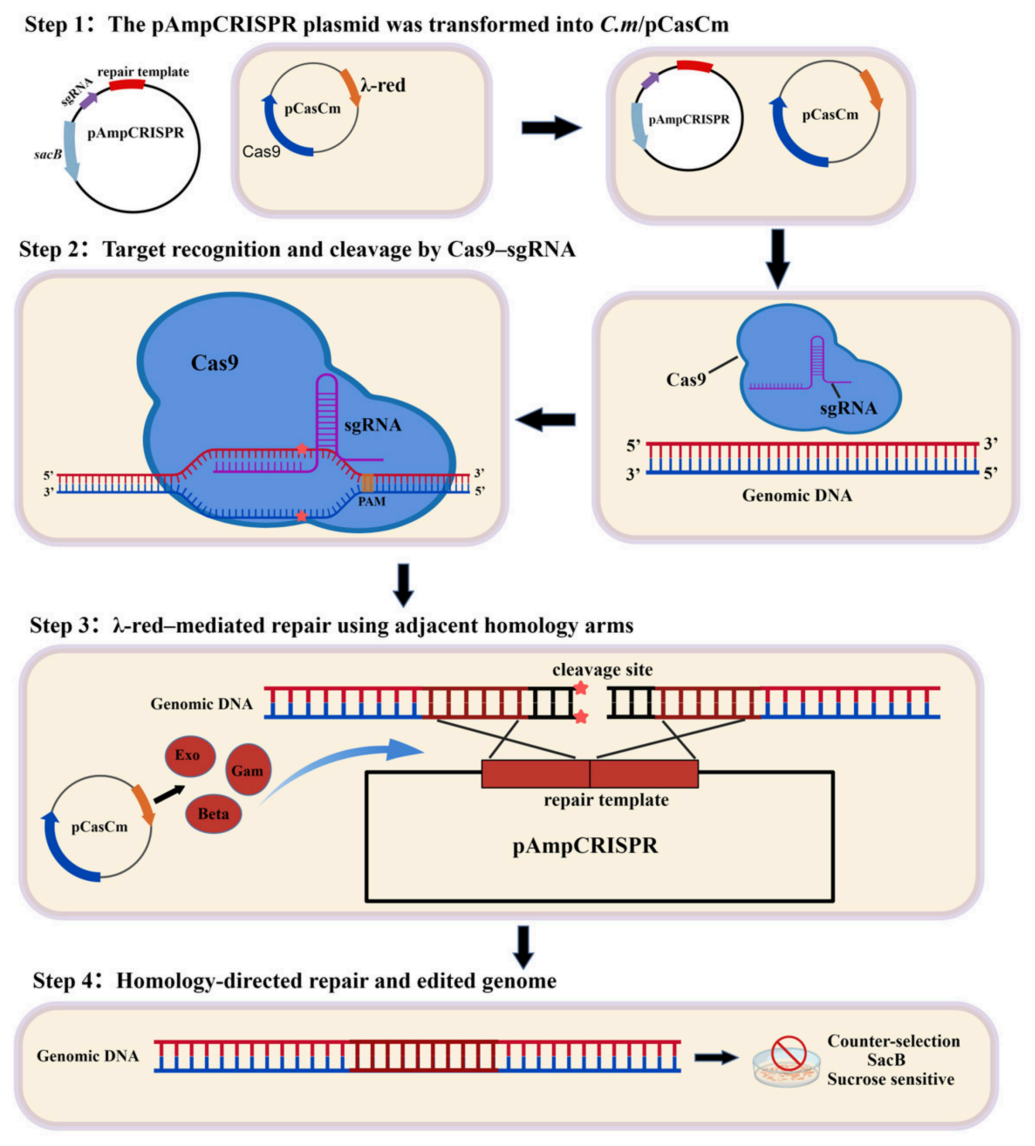
Schematic diagram of genome editing mediated by the pAmpCRISPR/pCasCm dual-plasmid system. The pAmpCRISPR plasmid carries the sgRNA, the *sacB* counter-selection marker, and adjacent homology arms (repair template), while pCasCm expresses Cas9 and the λ-Red recombination proteins (Exo, Beta, and Gam). Following transformation into a wild-type strain harboring pCasCm (*C.m*/pCasCm), the Cas9–sgRNA complex introduces a double-strand break at the target locus. The break is repaired via λ-Red-mediated homologous recombination using the homology arms as a template, resulting in precise genome editing. Correctly edited strains are obtained through *sacB*-based counter-selection. Stars denote the Cas9 cleavage sites.

**Figure 3:**
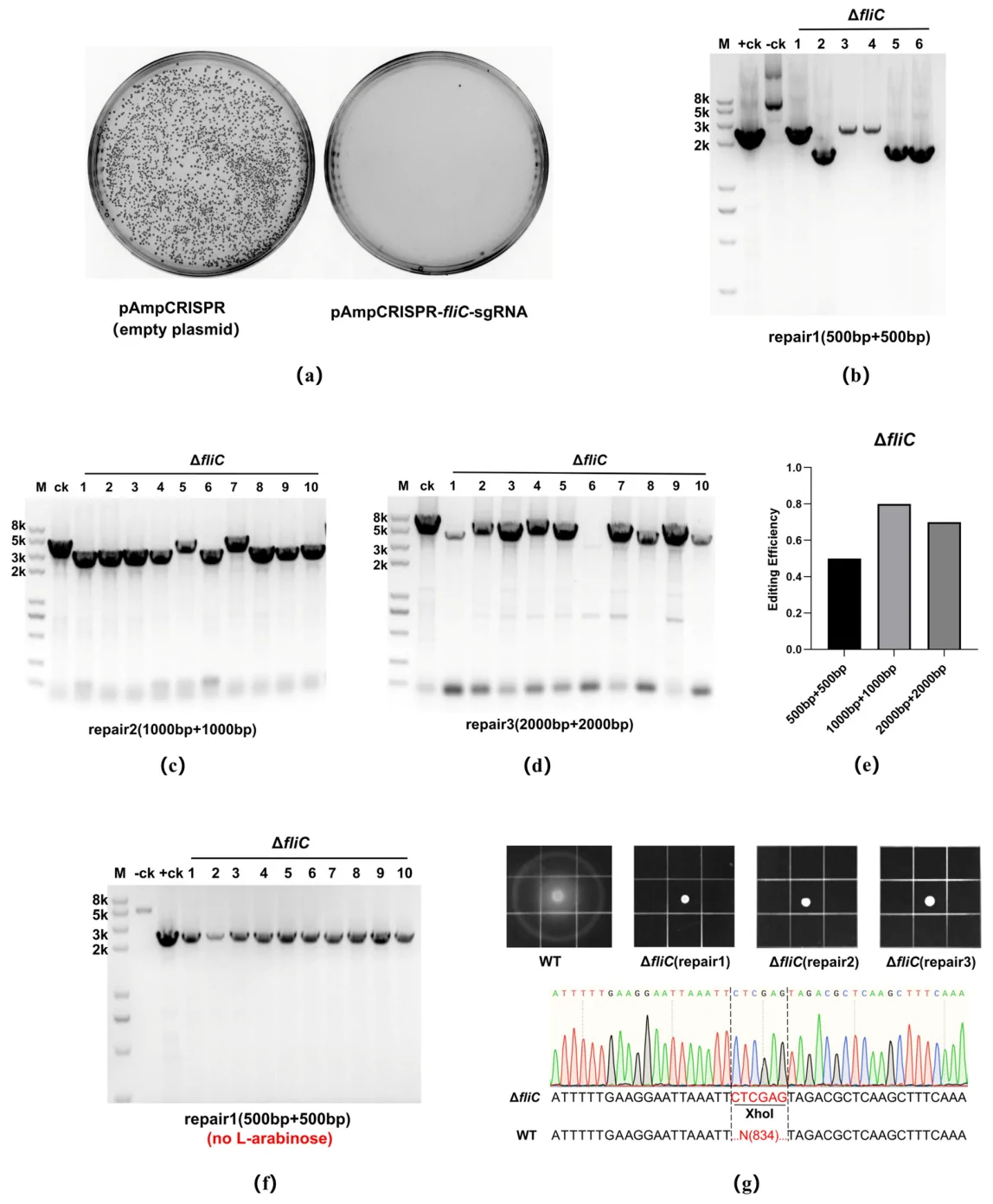
The pAmpCRISPR/pCasCm system enables efficient gene editing in *C. malonaticus*. (**a**) pAmpCRISPR (empty plasmid) and pAmpCRISPR-*fliC*-sgRNA carrying a 20-nt *fliC*-targeting sgRNA were electroporated into L-arabinose-induced *C.m*/pCasCm cells. Colony formation was observed after incubation on LB agar plates. (**b**) PCR verification of *fliC* deletion using a repair template containing 500 bp homology arms on each side in L-arabinose-induced *C.m*/pCasCm cells. (**c**) PCR verification of *fliC* deletion using a repair template containing 1000 bp homology arms on each side in L-arabinose-induced *C.m*/pCasCm cells. (**d**) PCR verification of *fliC* deletion using a repair template containing 2000 bp homology arms on each side in L-arabinose-induced *C.m*/pCasCm cells. (**e**) Comparison of *fliC* knockout outcomes using repair templates containing homology arms of different lengths on each side (500 bp, 1000 bp, 2000 bp). (**f**) PCR verification of *fliC* deletion using a repair template containing 500 bp homology arms on each side in uninduced *C.m*/pCasCm cells. (**g**) Motility of wild-type and Δ*fliC* strains was assessed on 0.3% semi-solid agar plates (representative images from three independent experiments). Confirmation of *fliC* deletion by DNA sequencing using the repair template containing 1000 bp homology arms on each side is shown below. +ck and ck, genomic DNA control; −ck, pAmpCRISPR-sgRNA-repair plasmid DNA control. The dashed lines indicate the sequence between the two homology arms in the wild-type and knockout strains.

To evaluate the editing efficiency of the pAmpCRISPR/pCasCm dual-plasmid system in *Cronobacter*, we first transformed the pCasCm plasmid into *C. malonaticus* (*C.m*/pCasCm). Following induction with L-arabinose, cells harboring pCasCm were harvested and prepared as electrocompetent cells. Subsequently, the empty pAmpCRISPR plasmid, pAmpCRISPR carrying a 20-nt *fliC*-targeting spacer (pAmpCRISPR-*fliC*-sgRNA), and pAmpCRISPR carrying both the spacer and homologous repair template (pAmpCRISPR-*fliC*-sgRNA-repair) were electroporated into *C.m*/pCasCm cells for genome editing (Figure 2). Transformation with the empty pAmpCRISPR plasmid yielded approximately 3000 single colonies (Figure 3a), corresponding to a transformation efficiency of 1.5 × 10^4^ CFU/μg. In contrast, transformation with pAmpCRISPR-*fliC*-sgRNA resulted in only one colony (Figure 3a), demonstrating that the *fliC*-targeting sgRNA directed Cas9 endonuclease to induce double-strand breaks at the *fliC* locus, leading to cell death. When pAmpCRISPR-*fliC*-sgRNA-repair containing 500 bp left and right homology arms was used, the observed deletion frequency for the *fliC* gene was five out of 10 colonies in a single trial (Figure 3b and Figure S1A). Given that homology arm length influences homologous recombination efficiency [22,26], we further tested repair templates containing 1000 bp or 2000 bp homology arms on each side together with the same 20-nt sgRNA. These modifications yielded eight out of 10 and seven out of 10 deletion colonies, respectively (Figure 3c,d and Figure S1A). Therefore, repair templates containing homology arms of 1000 bp or longer on each side achieved higher editing efficiencies than those with 500 bp homologous arms (Figure 3e), likely because longer homology arms facilitate homologous recombination.

The expression of the Cas9 nuclease and λ-Red recombination proteins is driven by the L-arabinose-inducible ParaB promoter. When pAmpCRISPR-*fliC*-sgRNA-repair carrying 500 bp homology arms on each side was electroporated into uninduced *C.m*/pCasCm electrocompetent cells, the transformed cells formed normal colonies on agar plates (Figure S1B). However, PCR analysis showed that *fliC* deletion was not achieved (Figure 3f), indicating that λ-Red-mediated homology recombination is essential for genome editing and that Cas9 activity requires L-arabinose induction.

As a Class III flagellar gene, deletion of *fliC* disrupts flagellar assembly, resulting in complete loss of bacterial motility [27]. To validate successful *fliC* knockout in *C. malonaticus*, we analyzed the motility phenotype of Δ*fliC* strains. Compared with the wild-type strain, Δ*fliC* mutants generated using homology arms of varying lengths completely lost motility on semi-solid agar plates (Figure 3g). Additionally, successful *fliC* knockout was further confirmed by DNA sequencing (Figure 3g).

In summary, these results demonstrated that the pAmpCRISPR/pCasCm dual-plasmid system enables efficient genome editing in *C. malonaticus*. With 1000 bp homology arms on each side used in this study, this system provides a useful tool for functional genomics research in *C. malonaticus*.

### 3.3. Application of pAmpCRISPR/pCasCm Dual-Plasmid System in C. malonaticus

**Figure 4:**
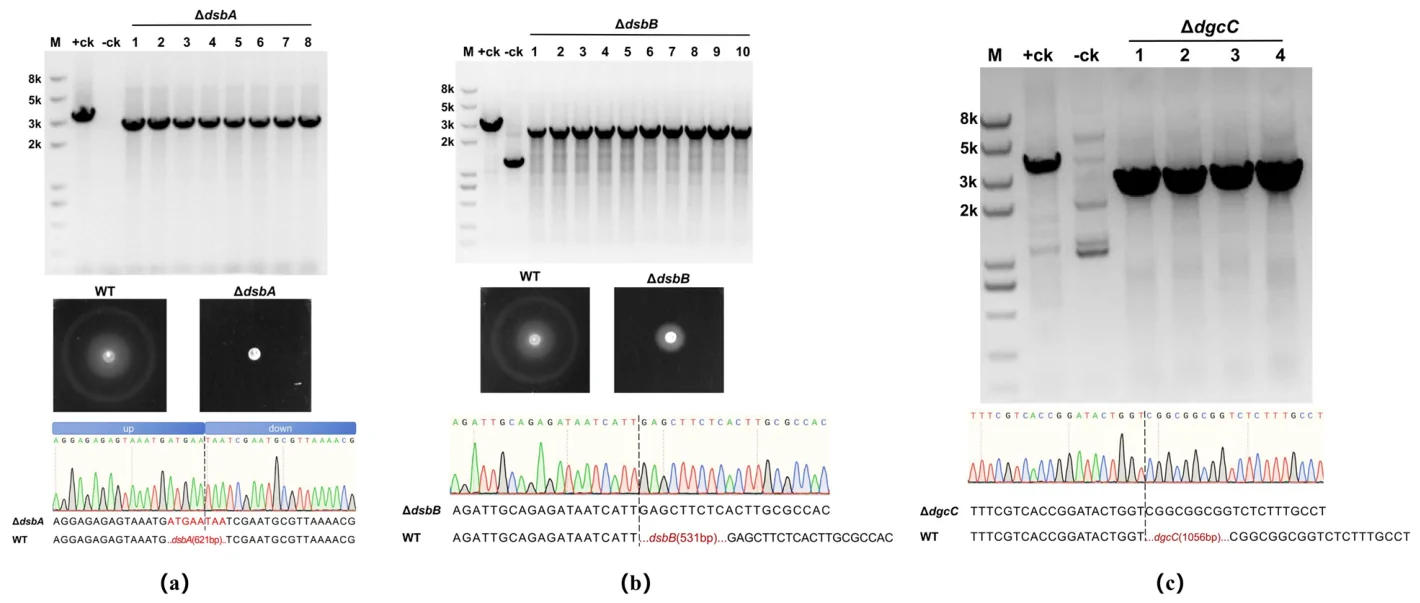
Using the pAmpCRISPR/pCasCm system and repair templates containing 1000 bp homology arms on each side, the *dsbA*, *dsbB*, and *dgcC* genes were efficiently deleted. (**a**) PCR verification of *dsbA* in *C. malonaticus* using the pAmpCRISPR/pCasCm system. Deletion was confirmed by PCR, DNA sequencing, and motility assays (representative images from three independent experiments). (**b**) PCR verification of *dsbB* in *C. malonaticus* using the pAmpCRISPR/pCasCm system. Deletion was confirmed by PCR, DNA sequencing, and motility assays (representative images from three independent experiments). (**c**) PCR verification of *dgcC* in *C. malonaticus* using the pAmpCRISPR/pCasCm system. Deletion was confirmed by PCR and DNA sequencing. +ck, genomic DNA control; −ck, pAmpCRISPR-sgRNA-repair plasmid DNA control. The dashed lines indicate the sequence between the two homology arms in the wild-type and knockout strains.

To evaluate the applicability of the pAmpCRISPR/pCasCm dual-plasmid system in *C. malonaticus*, we selected two genes (*dsbA* and *dgcC*) that failed to be knocked out using the traditional pCVD442-based suicide plasmid method (Figure S2), as well as another gene, *dsbB*. Recombinant pAmpCRISPR vectors carrying 20-nt sgRNA targeting each gene together with 1000 bp homology arms on each side were constructed and electroporated into *C.m*/pCasCm competent cells for genome editing. Knockout of *dsbA* and *dsbB* was successfully achieved, respectively (Figure 4a,b). In addition, motility and sequencing assays further confirmed successful gene deletion, as both Δ*dsbA* and Δ*dsbB* mutants exhibited reduced motility compared with the wild-type strain (Figure 4a,b). Although only four single colonies were obtained in the *dgcC* knockout experiment, PCR verification confirmed precise deletion of the target gene (Figure 4c). Successful deletion of *dgcC* was further validated by DNA sequencing (Figure 4c). These results suggest that the dual-plasmid system offers a useful alternative for complex genetic manipulations in *C. malonaticus*, particularly for loci recalcitrant to conventional methods.

### 3.4. Application of pAmpCRISPR/pCasCm Dual-Plasmid System for Exogenous Gene Cluster Integration

**Figure 5:**
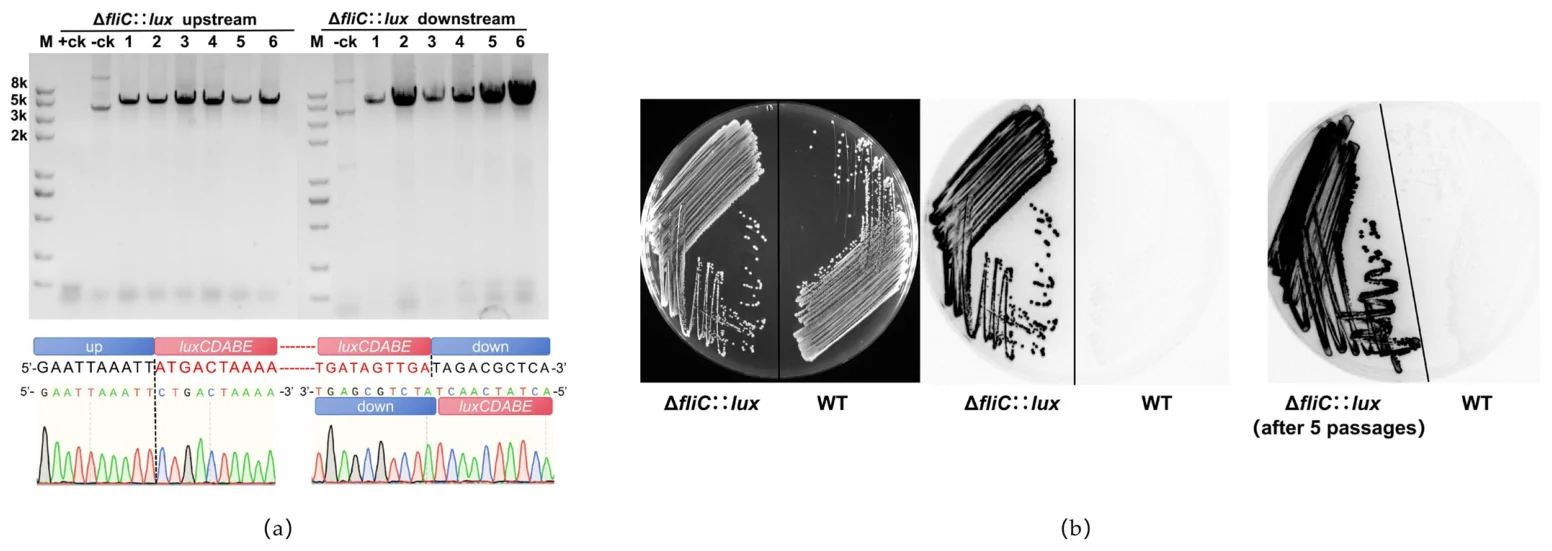
Large-fragment integration of *luxCDABE* mediated by the pAmpCRISPR/pCasCm system. (**a**) PCR and DNA sequencing verification of *luxCDABE* integration. (**Left**): upstream junction (*luxCDABE* ~2.8 kb + upstream homology arm ~2.0 kb); (**Right**): downstream junction (*luxCDABE* ~2.9 kb + downstream homology arm ~2.0 kb). DNA sequencing further confirmed precise integration of the *luxCDABE* cluster into the Δ*fliC* genome. +ck, genomic DNA control; −ck, pAmpCRISPR-sgRNA*^fliC^*-repair*^luxCDABE^* plasmid DNA control. (**b**) Images of the WT and Δ*fliC*∷*luxCDABE* strains captured under white-light illumination (**left**) and bioluminescence imaging conditions (**right**) using a gel imaging system. The *luxCDABE*-integrated strain retained stable bioluminescence after successive passages. The dashed lines indicate the sequence between the two homology arms in the wild-type and knockout strains.

To systematically evaluate the capacity of the pAmpCRISPR/pCasCm dual-plasmid system for integration and functional expression of exogenous gene clusters in *C. malonaticus*, we employed the *lux* bioluminescence reporter system as a functional reporter tool [28]. The 5798 bp *luxC*, *luxD, luxA*, *luxB*, *luxE* gene cluster (*luxCDABE*) was inserted between two 1000 bp homology arms flanking the target *fliC* locus, generating the plasmid pAmpCRISPR-sgRNA*^fliC^*-repair*^luxCDABE^*. This plasmid was subsequently electroporated into *C.m*/pCasCm cells. Colony PCR confirmed successful integration of the *luxCDABE* cluster and deletion of the *fliC* gene in the wild-type genome (Figure 5a and Figure S3). DNA sequencing further confirmed precise integration of the *luxCDABE* cluster into the Δ*fliC* genome (Figure 5a). Bioluminescence imaging revealed significantly enhanced bioluminescence in strains harboring the *luxCDABE* cluster compared with the wild-type strain (Figure 5b), demonstrating successful functional expression of the exogenous gene cluster at the chromosomal target site. Moreover, the *luxCDABE*-integrated strain retained stable bioluminescence after five successive passages, indicating stable maintenance and functional expression of the integrated *luxCDABE* cluster (Figure 5b). These results demonstrated that the pAmpCRISPR/pCasCm system not only enables gene knockout but also facilitates efficient integration of large exogenous gene clusters.

### 3.5. Application of the pAmpCRISPR/pCasCm Dual-Plasmid System in C. sakazakii

**Figure 6:**
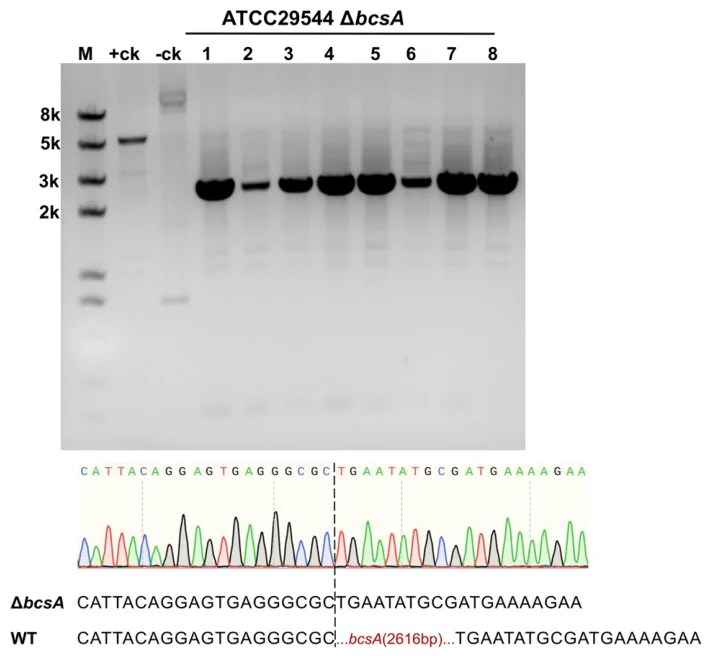
Deletion of *bcsA* in *C. sakazakii* ATCC 29544. Deletion of *bcsA* in *C. sakazakii* ATCC 29544 using the pAmpCRISPR/pCasCm system and repair templates containing 1000 bp homology arms on each side. Positive colonies were confirmed by PCR and DNA sequencing. +ck, genomic DNA control; −ck, pAmpCRISPR-sgRNA-*bcsA*-repair plasmid DNA control. The dashed lines indicate the sequence between the two homology arms in the wild-type and knockout strains.

To evaluate the broader applicability of the dual-plasmid system, we extended its use to another representative *Cronobacter* species, *C. sakazakii*. The pAmpCRISPR and pCasCm plasmids were electroporated into wild-type *C. sakazakii* cells. Following antibiotic selection and plasmid stability assays, both plasmids were confirmed to stably replicate in *C. sakazakii* without affecting bacterial growth. We next selected *bcsA*, a key gene involved in cellulose synthesis, as the target for knockout and designed corresponding homology arms for genome editing. PCR verification revealed gene-specific deletion bands in all eight randomly selected single colonies; DNA sequencing further confirmed precise deletion of the *bcsA* gene (Figure 6). These results suggest that the pAmpCRISPR/pCasCm dual-plasmid system has potential utility for precise and efficient gene editing in *C. sakazakii*.

## 4. Discussion

The advancement of functional genomics in under-characterized bacterial pathogens is critically dependent on the development of versatile and efficient genome editing tools. In this study, we established and optimized a CRISPR-Cas9-based dual-plasmid system (pAmpCRISPR/pCasCm) for precise genome editing in *C. malonaticus*. This system, which integrates CRISPR/Cas9 with the λ-Red homologous recombination system, has proven highly effective for both targeted gene knockouts and the seamless integration of large exogenous DNA fragments, thereby addressing a significant methodological gap in *Cronobacter* research.

The foundational principle of combining CRISPR-Cas9 with recombineering has been successfully validated across diverse bacteria. Pioneering work in *E. coli* established platforms using CRISPR-Cas9 to counter-select against wild-type cells, drastically improving recombination efficiency [19]. Similarly, adapted systems have been implemented in Salmonella, Staphylococcus aureus, and *Pseudomonas aeruginosa* for virulence studies and metabolic engineering, demonstrating the technology’s broad utility [21,22,29]. A key operational insight from these studies is that pre-expression of the λ-Red recombinases prior to CRISPR-mediated DNA cleavage significantly enhances editing success [19,30]. Our dual-plasmid strategy incorporates this principle by using a single plasmid (pCasCm) for the inducible co-expression of Cas9 and λ-Red proteins before introducing the targeting sgRNA and repair template on a second plasmid (pAmpCRISPR). This design aligns with established best practices and likely contributes to the high efficiency we observed.

The pAmpCRISPR/pCasCm system represents a useful supplementary toolkit for genetic manipulations in *Cronobacter*. Previously, genetic manipulations in this genus have relied predominantly on λ-Red-mediated recombination or suicide plasmid (e.g., pCVD442)-based systems [31,32,33]. While useful, these methods have notable limitations: the λ-Red method often leaves antibiotic marker scars, and the suicide plasmid method is notoriously time-consuming and labor-intensive. Additionally, pCVD442-based allelic exchange failed to disrupt certain loci (e.g., *dsbA* and *dgcC*), indicating locus-dependent applicability. In this study, the CRISPR-Cas9 system enables seamless, marker-free gene editing at the tested loci, including genes refractory to the pCVD442-based method, such as *dsbA* and *dgcC*. Furthermore, a single electroporation step suffices to generate mutants, significantly streamlining the workflow. The system’s utility is enhanced by features like the temperature-sensitive replicon and *sacB*-mediated counterselection, which facilitate the efficient curing of both plasmids to generate genetically stable, unmarked mutants [34]. However, the CRISPR-based system requires sequential cloning of sgRNA and homology arms, making construction more time-consuming than the single-step pCVD442 method. Beyond gene deletion, the capacity for stable chromosomal integration of large DNA fragments is crucial for mechanism research and metabolic engineering. Our system successfully facilitated the precise integration and functional expression of the ~5.8 kb *luxCDABE* bioluminescence gene cluster into the *C. malonaticus* genome. This demonstrates the system’s capability not only for knockout but also for sophisticated genome engineering, such as reporter strain construction or metabolic pathway implantation, expanding its application scope in this non-model organism.

The successful application of this system in *Cronobacter sakazakii*, where efficient knockout of the *bcsA* gene was achieved, suggests its potential applicability within the genus *Cronobacter*. This portability suggests that the pAmpCRISPR/pCasCm system can serve as a foundational genetic toolkit for the broader *Cronobacter* research community. Nevertheless, certain limitations provide avenues for future optimization. The editing efficiency showed a dependence on homology arm length, with 1000 bp arms yielding the highest efficiency among the lengths tested, consistent with known recombination kinetics [34]. Although these efficiency estimates were derived from a single independent experiment, additional replicates would further strengthen their robustness. Additionally, the requirement for L-arabinose induction adds a step, and the variable efficiency observed with different sgRNAs (e.g., for *dgcC*) highlights the need for careful sgRNA design and potential target-site dependency. This could be due to differences in sgRNA on-target activity or local DNA accessibility and suggests that designing multiple sgRNAs per target would improve robustness. Furthermore, while the system showed high reliability under our experimental conditions, we acknowledge that the number of tested loci and species was limited. The observed efficiency warrants further validation across a broader range of genomic targets and bacterial strains in future studies.

In conclusion, we have developed a CRISPR-Cas9 genome editing platform for *Cronobacter* spp. This system serves as a useful supplement to traditional genetic methods by combining high efficiency, seamless editing, and the capacity for large fragment integration. It provides a useful technical platform that will accelerate fundamental research into *Cronobacter* pathogenesis. Future work will employ this system to investigate the roles of specific virulence factors in *Cronobacter* pathogenesis and environmental fitness.

## Data Availability

The original contributions presented in this study are included in the article/Supplementary Materials. Further inquiries can be directed to the corresponding authors.

## Supplementary Materials

The following supporting information can be downloaded at https://www.mdpi.com/article/10.3390/pathogens15090990/s1, Figure S1: Evaluation of *fliC* deletion and plasmid curing using the pAmpCRISPR/pCasCm system; Figure S2: PCR verification of *dsaA* and *dgcC* deletion via pCVD442 suicide plasmid; Figure S3: Verification of *luxCDABE* integration and plasmid curing; Table S1: Strains and plasmids used in this study; Table S2: Primers used in this study; Data S1: Nucleotide sequence of the pAmpCRISPR plasmid; Data S2: Nucleotide sequence of the pCasCm plasmid.
