# Supplementary material for: A Dual-Plasmid CRISPR/Cas9-Based Genome Editing System for Efficient Gene Knockout and Large DNA Integration in Cronobacter malonaticus
Source: Pathogens. 2026 Sep 17;15(9):990. doi: 10.3390/pathogens15090990 (PMC13610606; doi:10.3390/pathogens15090990)
Supplement: Supplementary file 1 [file pathogens-15-00990-s001.zip › pathogens-4549666-supplementary.pdf]

Supplemental Information

Detailed workflow for gene editing mediated by the pAmpCRISPR/pCasCm dual-plasmid system.

(1) Spacer primer design

sgRNA sequences were designed using the CHOPCHOP web tool (https://chopchop.cbu.uib.no/). First, the target gene sequence was submitted to the platform, followed by selection of the corresponding species-specific genome database. The top-ranked sgRNA sequence with the highest predicted efficiency and lowest off-target potential was selected. Based on the CRISPR/Cas9 PAM requirement， the PAM sequences (NGG) at the 3' end was removed from the selected target sequence. Subsequently, "GTGG" was added to the 5' end to generate the forward oligonucleotide (sgRNA-F). For the reverse oligonucleotide (sgRNA-R), the reverse complementary sequences was generated, and "AAAC" was added to the 5' end.

(2) Phosphorylation of the oligos

Component	Volume
10× T4 DNA ligase buffer (Takara)	5 μL
50 μM Oligo F	2 μL
50 μM Oligo R	2 μL
T4 polynucleotide kinase (Takara)	1 μL
ddH_2O	40 μL
Total volume	50 μL

Incubation at 37 °C for 1 hour

(3) Annealing

2.5 μL of 1 M NaCl was added to the phosphorylated oligonucleotides. The phosphorylated oligonucleotides were then incubated at 95 °C for 3 min and gradually cooled to room temperature. Finally, sterile ddH_2O was added to dilute the annealed

oligonucleotides 20-fold to a final concentration of 100 nM.

(4) Golden Gate assembly（GGA）

Component	Volume
10×T4 DNA ligase buffer	1 μL
Annealed oligos (100 nM)	1 μL
The pACRISPR plasmid (20 nM)	1 μL
T4 DNA ligase	0.5 μL
Esp3I/BsmB-v2 (NEB)	0.5 μL
ddH_2O	6 μL
Total volume	10 μL

The PCR program was set as follows:

Segment	Temperature	Time	Cycles
1	37 °C	3 min	25
	16 °C	4 min	
2	80 °C	15 min	1
3	10 °C	5 min	1

(5) Transformation

The ligation product was transformed into chemically competent *Escherichia coli* DH5α cells. The transformed cells were plated on LB agar plates containing 100 μg/mL carbenicillin and incubated at 37 °C overnight. Positive colonies carrying the pAmpCRISPR-sgRNA plasmid were identified by colony PCR.

(6) Restriction digestion

The pAmpCRISPR-sgRNA plasmid was digested with the corresponding restriction enzymes to generate a linearized vector, which was subsequently assembled with the target DNA fragment by Gibson assembly (seamless cloning).

Component	Volume
Buffer	5 μL
2 μg pAmpCRISPR-sgRNA	xx μL
Enzyme 1	1.5 μL
Enzyme 2	1.5 μL
ddH_2O	xx μL
Total volume	50 μL

The reaction mixture was incubated at 37 °C for 2 ~ 3 h. The digested plasmid was purified using a PCR purification kit.

(7) Connection

The upstream and downstream homologous arm fragments of the target gene, amplified from genomic DNA, were fused using overlap PCR to generate a complete repair template. This template was then inserted into the restriction enzyme-digested pAmpCRISPR-sgRNA plasmid via either restriction-ligation or Gibson assembly. Finally, colony PCR was performed to screen recombinant pAmpCRISPR-sgRNA-H1+H2 plasmids with correctly integrated homologous arms at both ends.

(8) Repeat step (5) to construct the pAmpCRISPR-sgRNA-repair plasmid.

(9) Gene editing was performed using the pAmpCRISPR / pCasCm dual-plasmid system.

After preparation of electrocompetent cells harboring the pCasCm plasmid (*C.m*/pCasCm) as described in Materials and Methods. The pAmpCRISPR-sgRNA-repair plasmid was introduced into the competent cells. Then, cells were recovered at 30 °C for 2 h with shaking at 180 rpm and then plated on LB plates containing tetracycline (10 μg/mL) and crabenicillin (100 μg/mL), followed by incubation at 30 °C for 24-48 h to select for positive colonies. Individual colonies were picked and verified by colony PCR using gene-specific primers.

(10) The plasmids were cured after genome editing

To cure both plasmids after editing, the PCR-confirmed strains were inoculated into LB broth and incubated overnight at 37 °C with shaking. The overnight cultures were diluted and spread onto NaCl-free LB agar plates containing 10% sucrose, followed by incubation at 42 °C overnight. Colonies randomly selected from the sucrose-containing plates were streaked onto LB agar plates containing tetracycline (10 μg/mL), carbenicillin (100 μg/mL), and no antibiotics. The inability of the strains to grow on antibiotic-containing plates, together with normal growth on antibiotic-free plates, indicated that both the pAmpCRISPR and pCasCm plasmids had been successfully cured.

Table S1. Strains and plasmids used in this study.

Strains or Plasmids	Description	Sources
**Strains**		
DH5α	F-φ80,lacZΔM15,Δ(lacZYA-argF)U169endA1,recA1,hsdR(r_k-,m_k+)supE44,λ-thi-1,gyrA96,relA1,phoA	Invitrogen
C.malonaticus	Wild type	This study
C.sakazakii	Wild type	This study
**Plasmids**		
pEX18Tc	Tc^R，oriT$^+$sacB$^+$gene replacement vector with MCS；*sacB*，Tc^R	This lab
pKD46	Basic skeleton of pCasCm；Amp^R	This lab
pCasSA	Cas9；sgRNA	This lab
pAmpCRISPR	SacB；sgRNA；Amp^R；*oriT*	This lab
pCasCm	λRed；Cas9；Tc^R；pSC101	This study
pUCP20	*Escherichia coli* shuttle plasmid	This lab
pUCP20T	pUCP20 derived plasmid without Esp3I;*oriT*;Amp^R	This study

Table S2. Primers used in this study.

Primer	Sequence(5'→3'）	Application
sacB-F	CGGCATCAGAGCAGATTGTACT	For constructing pAmpCRISPR
sacB-R	GCAACTTTATGCCCATGCAAC	
sgRNA-F	TGGCGAGACGATTCGTCTCAGTTTTAGAGCTAGAAATAGCAAGTTAAAAT	
sgRNA-R	ATTGTTATCCGCTCACAATTAAAAAAAGCACCGACTCGGT	
tac-spacer-F	GAGCTGTTGACAATTAATCATCGGCTCGTATAATGTGTGGCGAGACGATTCGTCTC	
20T-F	AATTGTGAGCGGATAACAATTTC	For constructing pUCP20T
20T-R	GGTGCCTAATGAGTGAGCTAACTC	
18TC-*oriT*-F	ATAACGCAGGAAAGAACATGGGGATTCCTTAAGGTATACTTTCCGCTGCATAACCCTGC	
18TC-*oriT*-R	CTTTTGCTGGCCTTTTGCTCACATAAGCTAGCTTATCGGCCAGCCTCGCAGAGC	
pKD46-F	ACTCTTCCTTTTTCAATATTATTGAA	For constructing pCasCm
pKD46-R	CTGTCAGACCAAGTTTACTCATATATAC	
P_{araB}-cas-F	GTATATATGAGTAAACTTGGTCTGACAGAAGAAACCAATTGTCCATATTGC	
P_{araB}-cas-R	TCAGTCACCTCCTAGCTGACTC	
tetR-F	GAGTCAGCTAGGAGGTGACTGATGAAGCCAGTTACCTTCGG	For constructing pCasCm
tetR-R	TTCAATAATATTGAAAAAGGAAGAGTATGAA	

Primer	Sequence(5'→3')	Application
	ATCTAACAATGCGCTCA	
fliC-H1F-500	ACAGCTATGACCATGATTACGAATTCCGCTTCCAGCTTGTCATACA	For constructing pAmpCRISPR-*fliC*-sgRNA-repair1
fliC-H1R-500	TTTTGAAAGCTTGAGCGTCTAAATTTAATTCCTTCAAAAATTGG	
fliC-H2F-500	CCAATTTTTGAAGGAATTAAATTTAGACGCTCAAGCTTTCAAAA	
fliC-H2R-500	GTAAAACGACGGCCAGTGCCAAGCTTAATGCCTGGTTGGCAAGC	
yz-*fliC*-F	ACCACCACTTTGGCGTTCT	For constructing Δ*fliC*
fliC-H1F-1000	ACAGCTATGACCATGATTACGAATTCAGCCGATAATCCCCTGCA	For constructing pAmpCRISPR-*fliC*-sgRNA-repair2
fliC-H1R-1000	TTTTGAAAGCTTGAGCGTCTACTCGAGAATTTAATTCCTTCAAAAATTGG	
fliC-H2F-1000	CCAATTTTTGAAGGAATTAAATTCTCGAGTAGACGCTCAAGCTTTCAAAA	
fliC-H2R-1000	GCCTGCAGGTCGACTCTAGAGGATCCATCGCAGGAGACGAGTACC	
fliC-H1F-2000	ACAGCTATGACCATGATTACGAATTCTGTCGCCCTGTTCGATAAAC	For constructing pAmpCRISPR-*fliC*-sgRNA-repair3
fliC-H2R-2000	GCCTGCAGGTCGACTCTAGAGGATCCGATGTAGTCAAACTCGCCCG	
yz-*fliC*-F-2000	ACTCTTCCATTCTCGCCTGC	For constructing Δ*fliC*
lux-F	ATTTTTGAAGGAATTAAATTATGACTAAAAAAATTTCATTCATT	For constructing pAmpCRISPR-sgRNA^{fliC}-repair^{luxCDABE}
lux-H1R	CTTCCATATATCCCTCTGTCAT	
lux-H2F	ATGACAGAGGGATATATGGAAG	
lux-R	TTTGAAAGCTTGAGCGTCTATCAACTATCAAACGCTTCG	
fliC-sgRNA-F	GTGGGCTGTTGATACGCAGACCAG	For constructing pAmpCRISPR-*fliC*-sgRNA
fliC-sgRNA-R	AAACCTGGTCTGCGTATCAACAGC	
dsbA-sgRNA-F	GTGGGTTGGGTAAAGACCTGACGC	For constructing pAmpCRISPR-*dsbA*-sgRNA
dsbA-sgRNA-R	AAACGCGTCAGGTCTTTACCCAAC	
dsbA-H1F	ATTGGATCCCATTTATCAGTTAAAAATGCGTG	For constructing pAmpCRISPR-*dsbA*-sgRNA-repair
dsbA-H1R	TCGTTTTAACGCATTCGATTATTCATCATTTACTCTCTCCTGAAT	
dsbA-H2F	ATTCAGGAGAGAGTAAATGATGAATAATCGAATGCGTTAAAACGA	For constructing pAmpCRISPR-*dsbA*-sgRNA-repair
dsbA-H2R	ATTGGTACCATTATTTTTCGGCGGAAAAG	
yz-*dsbA*-F	ACATGTTTCCACAGCGCTTT	For constructing Δ*dsbA*

Primer	Sequence(5'→3')	Application
dsbB-H1-F	ATTGGATCCTTTCTGCGCAGTCTGATGATG	For constructing pAmpCRISPR-*dsbB*-sgRNA-repair
dsbB-H1-R	GTGGCGCAAGTGAGAAGCTCAATGATTATCTCTGCAATCTGCG	
dsbB-H2-F	CGCAGATTGCAGAGATAATCATTGAGCTTCTCACTTGCGCCAC	
dsbB-H2-R	ATTGGTACCTCCGGTATATCAGCTTTTGCG	
dsbB-sgRNA-F	GTGGCGTTACGCTTTGTCGCGATG	For constructing pAmpCRISPR-*dsbB*-sgRNA
dsbB-sgRNA-R	AAACCATCGCGACAAAGCGTAACG	
yz-*dsbB*-F	TTTTATGAAGCAGTTGCTGCTGC	For constructing Δ*dsbB*
dgcC-H1-F	ATGACCATGATTACGAATTCTGTCTGGCATTTCCCGTC	For constructing pAmpCRISPR-*dgcC*-sgRNA-repair
dgcC-H1-R	AAGAGACCGCCGCCGACCAGTATCCGGTGACGAAA	
dgcC-H2-F	TTTCGTCACCGGATACTGGTCGGCGGCGGTCTCTT	
dgcC-H2-R	AGGTCGACTCTAGAGGATCCTCTCGTTATCGGCGGCT	
dgcC-sgRNA-F	GTGGGAACGATCGCTACGGCCACG	For constructing pAmpCRISPR-*dgcC*-sgRNA
dgcC-sgRNA-F	AAACCGTGGCCGTAGCGATCGTTC	
yz-*dgcC*-F	TGTTTTATTTCCTGGGCGC	For constructing Δ*dgcC*
bcsA-sgRNA-F	GTGGGTGAACCTGCTGATGAAACG	For constructing pAmpCRISPR-*bcsA*-sgRNA
bcsA-sgRNA-R	AAACCGTTTCATCAGCAGGTTCAC	
bcsA-H1-F	ATTGGATCCATCATTAGCATGCAAACCGG	For constructing pAmpCRISPR-*dgcC*-sgRNA-repair
bcsA-H1-R	GTTTTCTTTTCATCGCATATTCAGCGCCCTCACTCCTGTAAT	
bcsA-H2-F	ATTACAGGAGTGAGGGCGCTGAATATGCGATGAAAAGAAAAC	
bcsA-H2-R	ATTAAGCTTTGGCTCATCATGCGTACCA	
yz-*bcsA*-F	GTGGTGATATGGATGACGGAT	For constructing Δ*bcsA*
pAmp-ver-F	TTCAACATTTCCGTGTCGCC	Verification of pAmpCRISPR plasmid curing by PCR
pAmp-ver-R	TGCAAGGCGATTAAGTTGGGT	
pCasCm-ver-F	ACGGCCAGTTAAGCCATTCAT	Verification of pCasCm plasmid curing by PCR
pCasCm-ver-R	GTGCCTGATGGTCGGAGACAA	
fliC-F2	GGGAAATAGCAGCCAGAATTTATT	Verification of *luxCDABE* gene cluster integration by PCR
fliC-R2	GGAAGCTTTCCTGAAAGGGTAAC	

Figure S1.

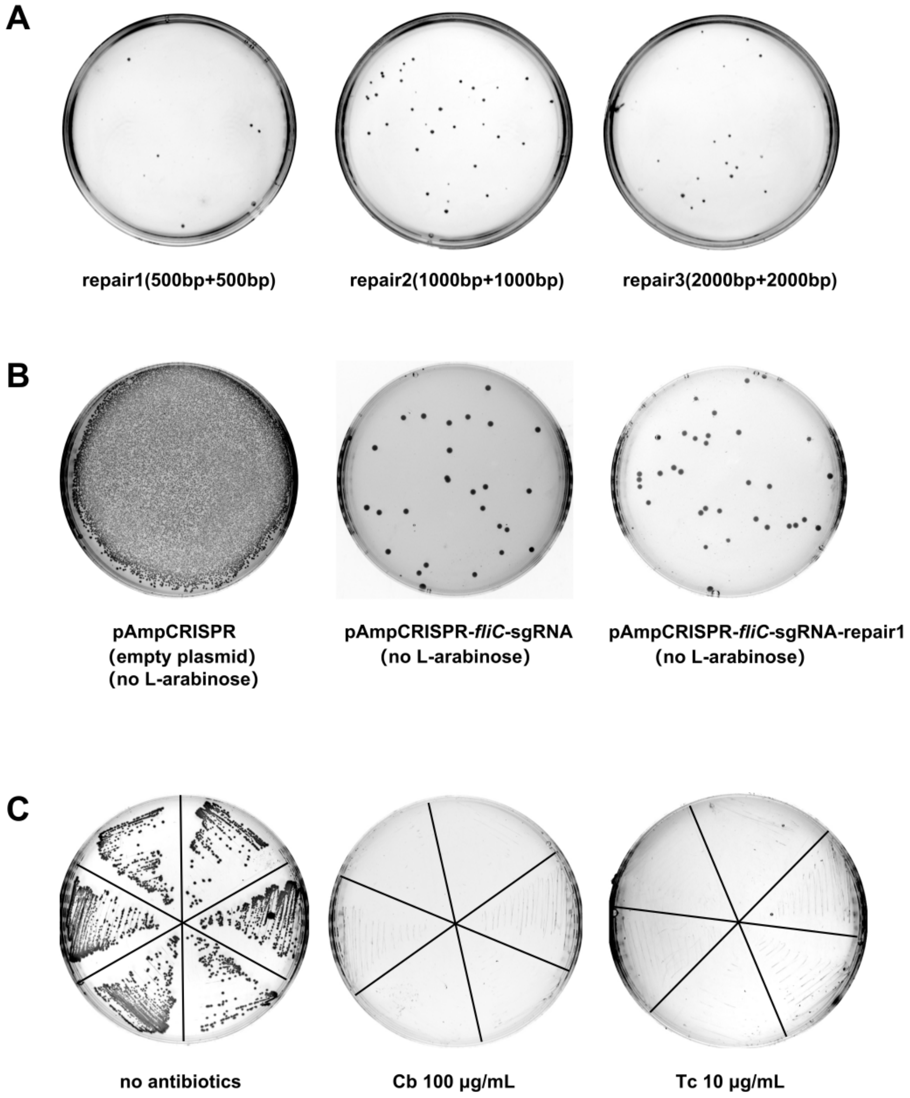

Figure S1. Evaluation of *fliC* deletion and plasmid curing using the pAmpCRISPR/pCasCm system.

(A) pAmpCRISPR-sgRNA-repair plasmids carrying repair templates of different lengths [500 bp (left), 1000 bp (middle), and 2000 bp (right)] were introduced by electroporation into L-arabinose–induced *C.m*/pCasCm strains for *fliC* gene deletion, and colony growth on LB agar plates containing carbenicillin (100 μg/mL) and tetracycline (10 μg/mL) was examined.

(B) pAmpCRISPR (empty plasmid), pAmpCRISPR-*fliC*-sgRNA (carrying a 20-nt *fliC*-targeting sgRNA), and pAmpCRISPR-*fliC*-sgRNA-repair1 (containing a 500-bp homologous arm) were separately introduced by electroporation into *C.m*/pCasCm strains without L-arabinose induction, and colony growth was examined following incubation on LB agar plates containing carbenicillin and tetracycline.

(C) *fliC* deletion mutants isolated from sucrose plates were streaked onto LB agar plates containing 100 μg/mL carbenicillin and 10 μg/mL tetracycline, on which no colony growth was observed, whereas normal growth was observed on antibiotic-free LB agar plates, indicating that the pAmpCRISPR and pCasCm plasmids had been successfully cured.

Figure S2.

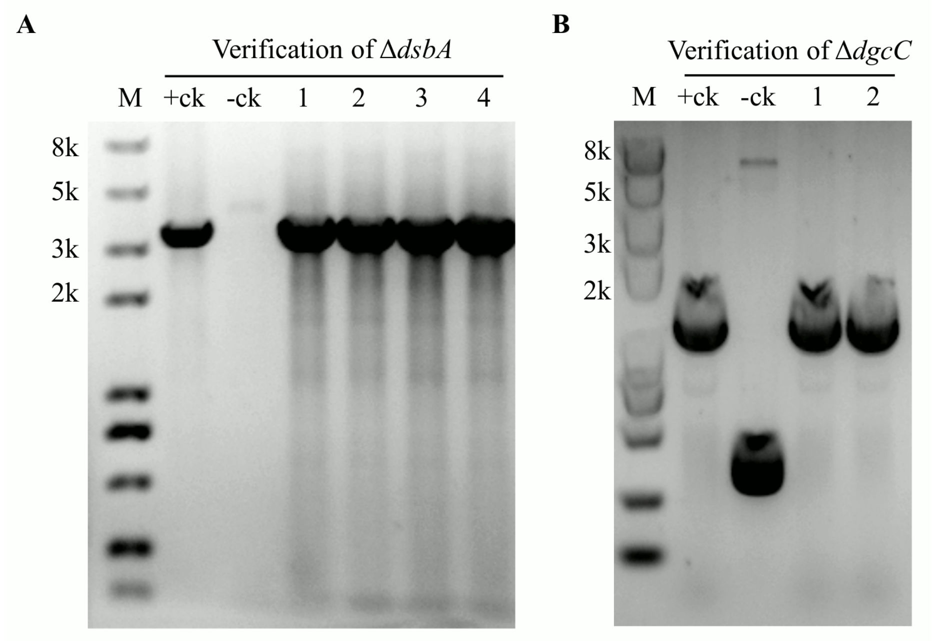

Figure S2. Verification of *dsbA* and *dgcC* deletion via pCVD442-based plasmids.

(A) PCR verification of *dsbA* deletion in *C. malonaticus*. M: marker; +ck: genome; -ck: suicide plasmid with homologous arms;

(B) PCR verification of *dgcC* deletion in *C. malonaticus*. M: marker; +ck: genome; -ck: suicide plasmid with homologous arms.

Figure S3.

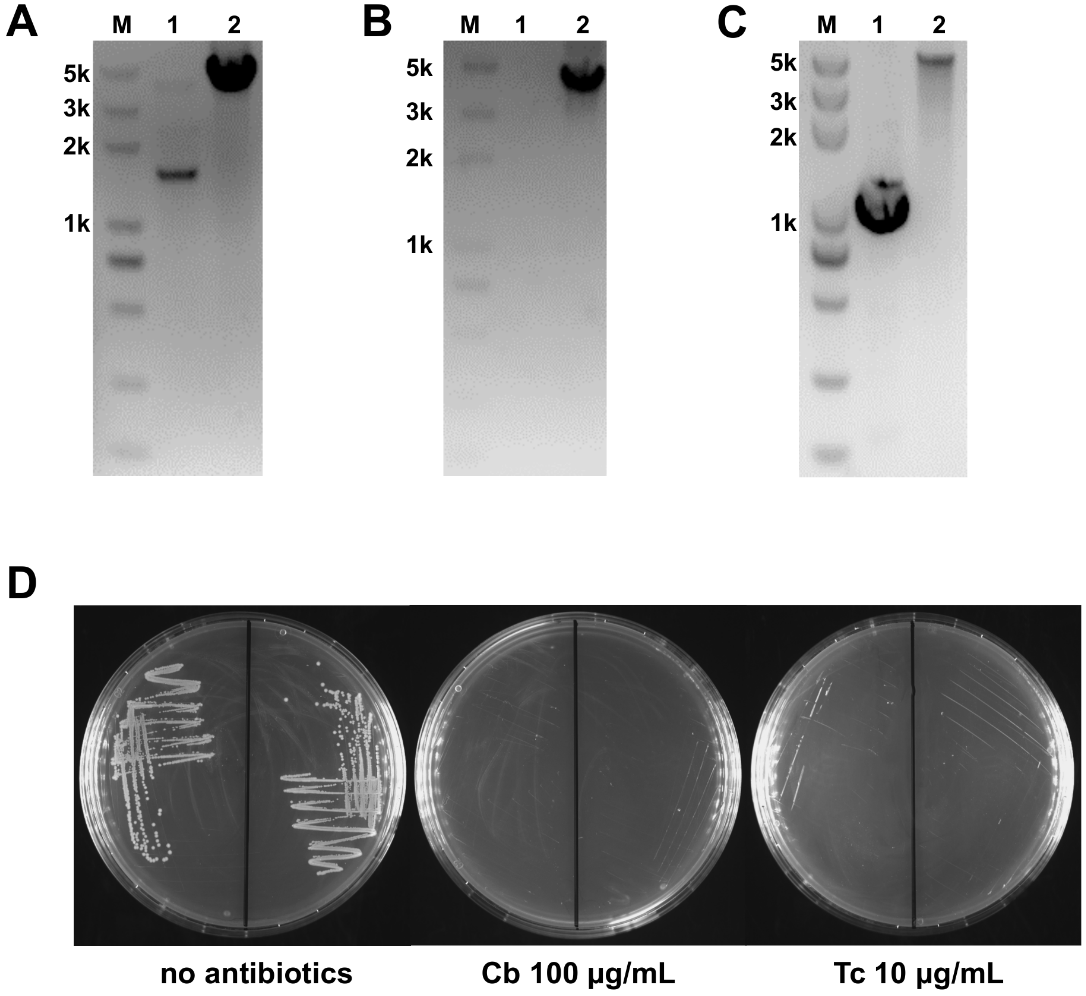

Figure S3. Verification of plasmid curing in the Δ*fliC*∷*luxCDABE* strain.

(A) PCR verification of successful curing of the pAmpCRISPR plasmid in the Δ*fliC*∷*luxCDABE* strain. M: marker; lane 1: the Δ*fliC*∷*luxCDABE* strain; lane 2: pAmpCRISPR plasmid, The expected size was 4,378 bp.

(B) PCR verification of successful curing of the pCasCm plasmid in the Δ*fliC*∷*luxCDABE* strain. M: marker; lane 1: the Δ*fliC*∷*luxCDABE* strain; lane 2: pCasCm plasmid, the expected size was 4,332 bp.

(C) Verification of *luxCDABE* gene cluster integration in the Δ*fliC*∷*luxCDABE* strain using primer pairs flanking *fliC* gene. M: marker; lane 1: the *C. malonaticus* wild-type strain; lanes 2: the Δ*fliC*∷*luxCDABE* strain, the expected amplicon size was 6,192 bp.

(D) Successful curing of the pAmpCRISPR and pCasCm plasmids. The Δ*fliC*∷*luxCDABE* strain colonies isolated from sucrose-containing plate were streaked onto LB agar plates supplemented with 100 μg/mL carbenicillin, 10 μg/mL tetracycline, or antibiotic-free LB agar plates, respectively.

Supplementary Data S1. Nucleotide sequence of the pAmpCRISPR plasmid.

>pAmpCRISPR

AAAGGGCCTCGTGATACGCCTATTTTTATAGGTTAATGTCATGATAATAATGGTTTCTTAGACGTCAGGT
GGCACTTTTCGGGGAAATGTGCGCGGAACCCCTATTTGTTTATTTTTCTAAATACATTCAAATATGTATC
CGCTCATGAGACAATAACCCTGATAAATGCTTCAATAATATTGAAAAAGGAAGAGTATGAGTATTCAAC
ATTTCCGTGTCGCCCTTATTCCCTTTTTTGCGGCATTTTGCCTTCCTGTTTTTGCTCACCCAGAAACGCT
GGTGAAAGTAAAAGATGCTGAAGATCAGTTGGGTGCACGAGTGGGTTACATCGAACTGGATCTCAAC
AGCGGTAAGATCCTTGAGAGTTTTCGCCCCGAAGAACGTTTTCCAATGATGAGCACTTTTAAAGTTCT
GCTATGTGGCGCGGTATTATCCCGTATTGACGCCGGGCAAGAGCAACTCGGTCGCCGCATACACTATTC
TCAGAATGACTTGGTTGAGTACTCACCAGTCACAGAAAAGCATCTTACGGATGGCATGACAGTAAGAG
AATTATGCAGTGCTGCCATAACCATGAGTGATAACACTGCGGCCAACTTACTTCTGACAACGATCGGAG
GACCGAAGGAGCTAACCGCTTTTTTGCACAACATGGGGGATCATGTAACTCGCCTTGATCGTTGGGAA
CCGGAGCTGAATGAAGCCATACCAAACGACGAGCGTGACACCACGATGCCTGTAGCAATGGCAACAA
CGTTGCGCAAACTATTAACTGGCGAACTACTTACTCTAGCTTCCCGGCAACAATTAATAGACTGGATGG
AGGCGGATAAAGTTGCAGGACCACTTCTGCGCTCGGCCCTTCCGGCTGGCTGGTTTATTGCTGATAAAT
CTGGAGCCGGTGAGCGTGGGTCTCGCGGTATCATTGCAGCACTGGGGCCAGATGGTAAGCCCTCCCGT
ATCGTAGTTATCTACACGACGGGGAGTCAGGCAACTATGGATGAACGAAATAGACAGATCGCTGAGAT
AGGTGCCTCACTGATTAAGCATTGGTAACTGTCAGACCAAGTTTACTCATATATACTTTAGATTGATTTA
AAACTTCATTTTTAATTTAAAAGGATCTAGGTGAAGATCCTTTTTGATAATCTCATGACCAAAATCCCTT
AACGTGAGTTTTCGTTCCACTGAGCGTCAGACCCCGTAGAAAAGATCAAAGGATCTTCTTGAGATCCT
TTTTTTCTGCGCGTAATCTGCTGCTTGCAAACAAAAAAACCACCGCTACCAGCGGTGGTTTGTTTGCC
GGATCAAGAGCTACCAACTCTTTTTCCGAAGGTAACTGGCTTCAGCAGAGCGCAGATACCAAATACTG
TCCTTCTAGTGTAGCCGTAGTTAGGCCACCACTTCAAGAACTCTGTAGCACCGCCTACATACCTCGCTC
TGCTAATCCTGTTACCAGTGGCTGCTGCCAGTGGCGATAAGTCGTGTCTTACCGGGTTGGACTCAAGA
CGATAGTTACCGGATAAGGCGCAGCGGTCGGGCTGAACGGGGGGTTCGTGCACACAGCCCAGCTTGG
AGCGAACGACCTACACCGAACTGAGATACCTACAGCGTGAGCTATGAGAAAGCGCCACGCTTCCCGA
AGGGAGAAAGGCGGACAGGTATCCGGTAAGCGGCAGGGTCGGAACAGGAGAGCGCACGAGGGAGC
TTCCAGGGGGAAACGCCTGGTATCTTTATAGTCCTGTCGGGTTTCGCCACCTCTGACTTGAGCGTCGAT
TTTTGTGATGCTCGTCAGGGGGGCGGAGCCTATGGAAAAACGCCAGCAACGCGGCCTTTTTACGGTTC
CTGGCCTTTTGCTGGCCTTTTGCTCACATAAGCTAGCTTATCGGCCAGCCTCGCAGAGCAGGATTCCCG
TTGAGCACCGCCAGGTGCGAATAAGGGACAGTGAAGAAGGAACACCCGCTCGCGGGTGGGCCTACTT
CACCTATCCTGCCCGGCTGACGCCGTTGGATACACCAAGGAAAGTCTACACGAACCCTTTGGCAAAAT
CCTGTATATCGTGCGAAAAAGGATGGATATACCGAAAAAATCGCTATAATGACCCCGAAGCAGGGTTAT
GCAGCGGAAAGTATACCTTAAGGAATCCCCATGTTCTTTCCTGCGTTATCCCCTGATTCTGTGGATAACC
GTATTACCGCCTTTGAGTGAGCTGATACCGCTCGCCGCAGCCGAACGACCGAGCGCAGCGAGTCAGT
GAGCGAGGAAGCGGAAGAGCGCCCAATACGCAAACCGCCTCTCCCCGCGCGTTGGCCGATTCATTAA
TGCAGCTGGCACGACAGGTTTCCCGACTGGAAAGCGGGCAGTGAGCGCAACGCAATTAATGTGAGTT
AGCTCACTCATTAGGCACCCGGCATCAGAGCAGATTGTACTGAGAGTGCACCATAATCGGCATTTTCTT
TTGCGTTTTTATTTGTTAACTGTTAATTGTCCTTGTTCAAGGATGCTGTCTTTGACAACAGATGTTTTCTT
GCCTTTGATGTTCAGCAGGAAGCTAGGCGCAAACGTTGATTGTTTGTCTGCGTAGAATCCTCTGTTTGT
CATATAGCTTGTAATCACGACATTGTTTCCTTTCGCTTGAGGTACAGCGAAGTGTGAGTAAGTAAAGGT
TACATCGTTAGGATCAAGATCCATTTTTAACACAAGGCCAGTTTTGTTCAGCGGCTTGTATGGGCCAGT
TAAAGAATTAGAAACATAACCAAGCATGTAAATATCGTTAGACGTAATGCCGTCAATCGTCATTTTTGAT
CCGCGGGAGTCAGTGAACAGATACCATTTGCCGTTCATTTTAAAGACGTTCGCGCGTTCAATTTCATCT

GTTACTGTGTTAGATGCAATCAGCGGTTTCATCACTTTTTTCAGTGTGTAATCATCGTTTAGCTCAATCA
TACCGAGAGCGCCGTTTGCTAACTCAGCCGTGCGTTTTTTATCGCTTTGCAGAAGTTTTTGACTTTCTT
GACGGAAGAATGATGTGCTTTTGCCATAGTATGCTTTGTTAAATAAAGATTCTTCGCCTTGGTAGCCATC
TTCAGTTCCAGTGTTTGCTTCAAATACTAAGTATTTGTGGCCTTTATCTTCTACGTAGTGAGGATCTCTC
AGCGTATGGTTGTCGCCTGAGCTGTAGTTGCCTTCATCGATGAACTGCTGTACATTTTGATACGTTTTTC
CGTCACCGTCAAAGATTGATTTATAATCCTCTACACCGTTGATGTTCAAAGAGCTGTCTGATGCTGATAC
GTTAACTTGTGCAGTTGTCAGTGTTTGTTTGCCGTAATGTTTACCGGAGAAATCAGTGTAGAATAAACG
GATTTTTCCGTCAGATGTAAATGTGGCTGAACCTGACCATTCTTGTGTTTGGTCTTTTAGGATAGAATCA
TTTGCATCGAATTTGTCGCTGTCTTTAAAGACGCGGCCAGCGTTTTTCCAGCTGTCAATAGAAGTTTCG
CCGACTTTTTGATAGAACATGTAAATCGATGTGTCATCCGCATTTTTAGGATCTCCGGCTAATGCAAAGA
CGATGTGGTAGCCGTGATAGTTTGCGACAGTGCCGTCAGCGTTTTGTAATGGCCAGCTGTCCCAAACG
TCCAGGCCTTTTGCAGAAGAGATATTTTTAATTGTGGACGAATCGAACTCAGGAACTTGATATTTTTCA
TTTTTTTGCTGTTCAGGGATTTGCAGCATATCATGGCGTGTAATATGGGAAATGCCGTATGTTTCCTTATA
TGGCTTTTGGTTCGTTTCTTTCGCAAACGCTTGAGTTGCGCCTCCTGCCAGCAGTGCGGTAGTAAAGG
TTAATACTGTTGCTTGTTTTGCAAACTTTTTGATGTTCATCGTTCATGTCTCCTTTTTTATGTACTGTGTT
AGCGGTCTGCTTCTTCCAGCCCTCCTGTTTGAAGATGGCAAGTTAGTTACGCACAATAAAAAAAGACC
TAAAATATGTAAGGGGTGACGCCAAAGTATACACTTTGCCCTTTACACATTTTAGGTCTTGCCTGCTTTA
TCAGTAACAAACCCGCGCGATTTACTTTTCGACCTCATTCTATTAGACTCTCGTTTGGATTGCAACTGGT
CTATTTTCCTCTTTTGTTTGATAGAAAATCATAAAAGGATTTGCAGACTACGGGCCTAAAGAACTAAAA
AATCTATCTGTTTCTTTTCATTCTCTGTATTTTTTATAGTTTCTGTTGCATGGGCATAAAGTTGCGAGCTG
TTGACAATTAATCATCGGCTCGTATAATGTGTGGCGAGACGATTCGTCTCAGTTTTAGAGCTAGAAATA
GCAAGTTAAAATAAGGCTAGTCCGTTATCAACTTGAAAAAGTGGCACCGAGTCGGTGCTTTTTTTAATT
GTGAGCGGATAACAATTTCACACAGGAAACAGCTATGACCATGATTACGAATTCGAGCTCGGTACCCG
GGGATCCTCTAGAGTCGACCTGCAGGCATGCAAGCTTGGCACTGGCCGTCGTTTTACAACGTCGTGAC
TGGGAAAACCCTGGCGTTACCCAACTTAATCGCCTTGCAGCACATCCCCCTTTCGCCAGCTGGCGTAA
TAGCGAAGAGGCCCGCACCGATCGCCCTTCCCAACAGTTGCGCAGCCTGAAAGGCAGGCCGGGCCGT
GGTGGCCACGGCCTCTAGGCCAGATCCAGCGGCATCTGGGTTAGTCGAGCGCGGGCCGCTTCCCATGT
CTCACCAGGGCGAGCCTGTTTCGCGATCTCAGCATCTGAAATCTTCCCGGCCTTGCGCTTCGCTGGGG
CCTTACCCACCGCCTTGGCGGGCTTCTTCGGTCCAAAACTGAACAACAGATGTGTGACCTTGCGCCCG
GTCTTTCGCTGCGCCCACTCCACCTGTAGCGGGCTGTGCTCGTTGATCTGCGTCACGGCTGGATCAAG
CACTCGCAACTTGAAGTCCTTGATCGAGGGATACCGGCCTTCCAGTTGAAACCACTTTCGCAGCTGGT
CAATTTCTATTTCGCGCTGGCCGATGCTGTCCCATTGCATGAGCAGCTCGTAAAGCCTGATCGCGTGGG
TGCTGTCCATCTTGGCCACGTCAGCCAAGGCGTATTTGGTGAACTGTTTGGTGAGTTCCGTCAGGTAC
GGCAGCATGTCTTTGGTGAACCTGAGTTCTACACGGCCCTCACCCTCCCGGTAGATGATTGTTTGCACC
CAGCCGGTAATCATCACACTCGGTCTTTTCCCCTTGCCATTGGGCTCTTGGGTTAACCGGACTTCCCGC
CGTTTCAGGCGCAGGGCCGCTTCTTTGAGCTGGTTGTAGGAAGATTCGATAGGGACACCCGCCATCGT
CGCTATGTCCTCCGCCGTCACTGAATACATCACTTCATCGGTGACAGGCTCGCTCCTCTTCACCTGGCT
AATACAGGCCAGAACGATCCGCTGTTCCTGAACACTGAGGCGATACGCGGCCTCGACCAGGGCATTGC
TTTTGTAAACCATTGGGGGTGAGGCCACGTTCGACATTCCTTGTGTATAAGGGGACACTGTATCTGCGT
CCCACAATACAACAAATCCGTCCCTTTACAACAACAAATCCGTCCCTTCTTAACAACAAATCCGTCCCT
TAATGGCAACAAATCCGTCCCTTTTTAAACTCTACAGGCCACGGATTACGTGGCCTGTAGACGTCCTAA
AAGGTTTAAAAGGGAAAAGGAAGAAAAGGGTGGAAACGCAAAAAACGCACCACTACGTGGCCCCGT
TGGGGCCGCATTTGTGCCCCTGAAGGGGCGGGGGAGGCGTCTGGGCAATCCCCGTTTTACCAGTCCCC
TATCGCCGCCTGAGAGGGCGCAGGAAGCGAGTAATCAGGGTATCGAGGCGGATTCACCCTTGGCGTCC

AACCAGCGGCACCAGCGGCGCCTGAGAGGTATGGTGCACTCTCAGTACAATCTGCTCTGATGCCGCAT
AGTTAAGCCAGCCCCGACACCCGCCAACACCCGCTGACGCGCCCTGACGGGCTTGTCTGCTCCCGGC
ATCCGCTTACAGACAAGCTGTGACCGGGAGCTGCATGTGTCAGAGGTTTTCACCGTCATCACCGAAAC
GCGC

Supplementary Data S2. Nucleotide sequence of the pCasCm plasmid.

>pCasCm

CATCGATTTATTATGACAACTTGACGGCTACATCATTCACTTTTTCTTCACAACCGGCACGGAACTCGCT
CGGGCTGGCCCCGGTGCATTTTTTAAATACCCGCGAGAAATAGAGTTGATCGTCAAAACCAACATTGC
GACCGACGGTGGCGATAGGCATCCGGGTGGTGCTCAAAAGCAGCTTCGCCTGGCTGATACGTTGGTCC
TCGCGCCAGCTTAAGACGCTAATCCCTAACTGCTGGCGGAAAAGATGTGACAGACGCGACGGCGACA
AGCAAACATGCTGTGCGACGCTGGCGATATCAAAATTGCTGTCTGCCAGGTGATCGCTGATGTACTGA
CAAGCCTCGCGTACCCGATTATCCATCGGTGGATGGAGCGACTCGTTAATCGCTTCCATGCGCCGCAGT
AACAATTGCTCAAGCAGATTTATCGCCAGCAGCTCCGAATAGCGCCCTTCCCCTTGCCCGGCGTTAATG
ATTTGCCCAAACAGGTCGCTGAAATGCGGCTGGTGCGCTTCATCCGGGCGAAAGAACCCCGTATTGGC
AAATATTGACGGCCAGTTAAGCCATTCATGCCAGTAGGCGCGCGGACGAAAGTAAACCCACTGGTGAT
ACCATTCGCGAGCCTCCGGATGACGACCGTAGTGATGAATCTCTCCTGGCGGGAACAGCAAAATATCA
CCCGGTCGGCAAACAAATTCTCGTCCCTGATTTTTCACCACCCCCTGACCGCGAATGGTGAGATTGAG
AATATAACCTTTCATTCCCAGCGGTCGGTCGATAAAAAAATCGAGATAACCGTTGGCCTCAATCGGCGT
TAAACCCGCCACCAGATGGGCATTAAACGAGTATCCCGGCAGCAGGGGATCATTTTGCGCTTCAGCCA
TACTTTTCATACTCCCGCCATTCAGAGAAGAAACCAATTGTCCATATTGCATCAGACATTGCCGTCACT
GCGTCTTTTACTGGCTCTTCTCGCTAACCAAACCGGTAACCCCGCTTATTAAAAGCATTCTGTAACAAA
GCGGGACCAAAGCCATGACAAAAACGCGTAACAAAAGTGTCTATAATCACGGCAGAAAAGTCCACAT
TGATTATTTGCACGGCGTCACACTTTGCTATGCCATAGCATTTTTATCCATAAGATTAGCGGATCCTACCT
GACGCTTTTTATCGCAACTCTCTACTGTTTCTCCATACCCGTTTTTTTGGGAATTCGAGCTCTAAGGAGG
TTATAAAAAATGGATATTAATACTGAAACTGAGATCAAGCAAAAGCATTCACTAACCCCCTTTCCTGTT
TTCCTAATCAGCCCGGCATTTCGCGGGCGATATTTTCACAGCTATTTCAGGAGTTCAGCCATGAACGCT
TATTACATTCAGGATCGTCTTGAGGCTCAGAGCTGGGCGCGTCACTACCAGCAGCTCGCCCGTGAAGA
GAAAGAGGCAGAACTGGCAGACGACATGGAAAAAGGCCTGCCCCAGCACCTGTTTGAATCGCTATGC
ATCGATCATTTGCAACGCCACGGGGCCAGCAAAAAATCCATTACCCGTGCGTTTGATGACGATGTTGA
GTTTCAGGAGCGCATGGCAGAACACATCCGGTACATGGTTGAAACCATTGCTCACCACCAGGTTGATA
TTGATTCAGAGGTATAAAACGAATGAGTACTGCACTCGCAACGCTGGCTGGGAAGCTGGCTGAACGT
GTCGGCATGGATTCTGTCGACCCACAGGAACTGATCACCACTCTTCGCCAGACGGCATTTAAAGGTGA
TGCCAGCGATGCGCAGTTCATCGCATTACTGATCGTTGCCAACCAGTACGGCCTTAATCCGTGGACGA
AAGAAATTTACGCCTTTCCTGATAAGCAGAATGGCATCGTTCCGGTGGTGGGCGTTGATGGCTGGTCC
CGCATCATCAATGAAAACCAGCAGTTTGATGGCATGGACTTTGAGCAGGACAATGAATCCTGTACATG
CCGGATTTACCGCAAGGACCGTAATCATCCGATCTGCGTTACCGAATGGATGGATGAATGCCGCCGCGA
ACCATTCAAAACTCGCGAAGGCAGAGAAATCACGGGGCCGTGGCAGTCGCATCCCAAACGGATGTTA
CGTCATAAAGCCATGATTCAGTGTGCCCGTCTGGCCTTCGGATTTGCTGGTATCTATGACAAGGATGAA
GCCGAGCGCATTGTCGAAAATACTGCATACACTGCAGAACGTCAGCCGGAACGCGACATCACTCCGGT
TAACGATGAAACCATGCAGGAGATTAACACTCTGCTGATCGCCCTGGATAAAACATGGGATGACGACT
TATTGCCGCTCTGTTCCCAGATATTTCGCCGCGACATTCGTGCATCGTCAGAACTGACACAGGCCGAAG
CAGTAAAAGCTCTTGGATTCCTGAAACAGAAAGCCGCAGAGCAGAAGGTGGCAGCATGACACCGGA
CATTATCCTGCAGCGTACCGGGATCGATGTGAGAGCTGTCGAACAGGGGGATGATGCGTGGCACAAAT
TACGGCTCGGCGTCATCACCGCTTCAGAAGTTCACAACGTGATAGCAAAACCCCGCTCCGGAAAGAA
GTGGCCTGACATGAAAATGTCCTACTTCCACACCCTGCTTGCTGAGGTTTGCACCGGTGTGGCTCCGG
AAGTTAACGCTAAAGCACTGGCCTGGGGAAAACAGTACGAGAACGACGCCAGAACCCTGTTTGAATT
CACTTCCGGCGTGAATGTTACTGAATCCCCGATCATCTATCGCGACGAAAGTATGCGTACCGCCTGCTC
TCCCGATGGTTTATGCAGTGACGGCAACGGCCTTGAACTGAAATGCCCGTTTACCTCCCGGGATTTCAT

GAAGTTCCGGCTCGGTGGTTTCGAGGCCATAAAGTCAGCTTACATGGCCCAGGTGCAGTACAGCATGT
GGGTGACGCGAAAAAATGCCTGGTACTTTGCCAACTATGACCCGCGTATGAAGCGTGAAGGCCTGCAT
TATGTCGTGATTGAGCGGGATGAAAAGTACATGGCGAGTTTTGACGAGATCGTGCCGGAGTTCATCGA
AAAAATGGACGAGGCACTGGCTGAAATTGGTTTTGTATTTGGGGAGCAATGGCGATGACGCATCCTCA
CGATAATATCCGGGTAGGCGCAATCACTTTCGTCTACTCCGTTACAAAGCGAGGCTGGGTATTTCCCGG
CCTTTCTGTTATCCGAAATCCACTGAAAGCACAGCGGCTGGCTGAGGAGATAAATAATAAACGAGGGG
CTGTATGCACAAAGCATCTTCTGTTGAGTTAAGAACGAGTATCGAGATGGCACATAGCCTTGCTCAAAT
TGGAATCAGGTTTGTGCCAATACCAGTAGAAACAGACGAAGAATCCATGGGTATGGACAGTTTTCCCT
TTGATATGTAACGGTGAACAGTTGTTCTACTTTTGTTTGTTAGTCTTGATGCTTCACTGATAGATACAAG
AGCCATAAGAACCTCAGATCCTTCCGTATTTAGCCAGTATGTTCTCTAGTGTGGTTCGTTGTTTTTGCGT
GAGCCATGAGAACGAACCATTGAGATCATACTTACTTTGCATGTCACTCAAAAATTTTGCCTCAAAACT
GGTGAGCTGAATTTTTGCAGTTAAAGCATCGTGTAGTGTTTTTCTTAGTCCGTTACGTAGGTAGGAATC
TGATGTAATGGTTGTTGGTATTTTGTCACCATTCATTTTTATCTGGTTGTTCTCAAGTTCGGTTACGAGAT
CCATTTGTCTATCTAGTTCAACTTGGAAAATCAACGTATCAGTCGGGCGGCCTCGCTTATCAACCACCA
ATTTCATATTGCTGTAAGTGTTTAAATCTTTACTTATTGGTTTCAAAACCCATTGGTTAAGCCTTTTAAAC
TCATGGTAGTTATTTTCAAGCATTAACATGAACTTAAATTCATCAAGGCTAATCTCTATATTTGCCTTGTG
AGTTTTCTTTTGTGTTAGTTCTTTTAATAACCACTCATAAATCCTCATAGAGTATTTGTTTTCAAAAGACT
TAACATGTTCCAGATTATATTTTATGAATTTTTTTAACTGGAAAAGATAAGGCAATATCTCTTCACTAAAA
ACTAATTCTAATTTTTCGCTTGAGAACTTGGCATAGTTTGTCCACTGGAAAATCTCAAAGCCTTTAACC
AAAGGATTCCTGATTTCCACAGTTCTCGTCATCAGCTCTCTGGTTGCTTTAGCTAATACACCATAAGCAT
TTTCCCTACTGATGTTCATCATCTGAGCGTATTGGTTATAAGTGAACGATACCGTCCGTTCTTTCCTTGTA
GGGTTTTCAATCGTGGGGTTGAGTAGTGCCACACAGCATAAAATTAGCTTGGTTTCATGCTCCGTTAAG
TCATAGCGACTAATCGCTAGTTCATTTGCTTTGAAAACAACTAATTCAGACATACATCTCAATTGGTCTA
GGTGATTTTAATCACTATACCAATTGAGATGGGCTAGTCAATGATAATTACTAGTCCTTTTCCTTTGAGTT
GTGGGTATCTGTAAATTCTGCTAGACCTTTGCTGGAAAACTTGTAAATTCTGCTAGACCCTCTGTAAATT
CCGCTAGACCTTTGTGTGTTTTTTTTGTTTATATTCAAGTGGTTATAATTTATAGAATAAAGAAAGAATAA
AAAAAGATAAAAAGAATAGATCCCAGCCCTGTGTATAACTCACTACTTTAGTCAGTTCCGCAGTATTAC
AAAAGGATGTCGCAAACGCTGTTTGCTCCTCTACAAAACAGACCTTAAAACCCTAAAGGCTTAAGTAG
CACCCTCGCAAGCTCGGTTGCGGCCGCAATCGGGCAAATCGCTGAATATTCCTTTTGTCTCCGACCATC
AGGCACCTGAGTCGCTGTCTTTTTCGTGACATTCAGTTCGCTGCGCTCACGGCTCTGGCAGTGAATGG
GGGTAAATGGCACTACAGGCGCCTTTTATGGATTCATGCAAGGAAACTACCCATAATACAAGAAAAGC
CCGTCACGGGCTTCTCAGGGCGTTTTATGGCGGGTCTGCTATGTGGTGCTATCTGACTTTTTGCTGTTC
AGCAGTTCCTGCCCTCTGATTTTCCAGTCTGACCACTTCGGATTATCCCGTGACAGGTCATTCAGACTG
GCTAATGCACCCAGTAAGGCAGCGGTATCATCAACGGGGTCTGACGCTCAGTGGAACGAAAACTCAC
GTTAAGGGATTTTGGTCATGAGATTATCAAAAAGGATCTTCACCTAGATCCTTTTAAATTAAAAATGAA
GTTTTAAATCAATCTAAAGTATATATGAGTAAACTTGGTCTGACAGAAGAAACCAATTGTCCATATTGCA
TCAGACATTGCCGTCACTGCGTCTTTTACTGGCTCTTCTCGCTAACCAAACCGGTAACCCCGCTTATTA
AAAGCATTCTGTAACAAAGCGGGACCAAAGCCATGACAAAAACGCGTAACAAAAGTGTCTATAATCA
CGGCAGAAAAGTCCACATTGATTATTTGCACGGCGTCACACTTTGCTATGCCATAGCATTTTTATCCATA
AGATTAGCGGATCCTACCTGACGCTTTTTATCGCAACTCTCTACTGTTTCTCCATACCCGTTTTTTTGGG
AATTCGAGCTCTAAGGAGGTTATAAAAAATGGATAAGAAATACTCAATAGGCTTAGATATCGGCACAAA
TAGCGTCGGATGGGCGGTGATCACTGATGAATATAAGGTTCCGTCTAAAAAGTTCAAGGTTCTGGGAA
ATACAGACCGCCACAGTATCAAAAAAAATCTTATAGGGGCTCTTTTATTTGACAGTGGAGAGACAGCG
GAAGCGACTCGTCTCAAACGGACAGCTCGTAGAAGGTATACACGTCGGAAGAATCGTATTTGTTATCT

ACAGGAGATTTTTTCAAATGAGATGGCGAAAGTAGATGATAGTTTCTTTCATCGACTTGAAGAGTCTTT
TTTGGTGGAAGAAGACAAGAAGCATGAACGTCATCCTATTTTTGGAAATATAGTAGATGAAGTTGCTTA
TCATGAGAAATATCCAACTATCTATCATCTGCGAAAAAAATTGGTAGATTCTACTGATAAAGCGGATTTG
CGCTTAATCTATTTGGCCTTAGCGCATATGATTAAGTTTCGTGGTCATTTTTTGATTGAGGGAGATTTAA
ATCCTGATAATAGTGATGTGGACAAACTATTTATCCAGTTGGTACAAACCTACAATCAATTATTTGAAGA
AAACCCTATTAACGCAAGTGGAGTAGATGCTAAAGCGATTCTTTCTGCACGATTGAGTAAATCAAGAC
GATTAGAAAATCTCATTGCTCAGCTCCCCGGTGAGAAGAAAAATGGCTTATTTGGGAATCTCATTGCTT
TGTCATTGGGTTTGACCCCTAATTTTAAATCAAATTTTGATTTGGCAGAAGATGCTAAATTACAGCTTTC
AAAAGATACTTACGATGATGATTTAGATAATTTATTGGCGCAAATTGGAGATCAATATGCTGATTTGTTTT
TGGCAGCTAAGAATTTATCAGATGCTATTTTACTTTCAGATATCCTAAGAGTAAATACTGAAATAACTAA
GGCTCCCCTATCAGCTTCAATGATTAAACGCTACGATGAACATCATCAAGACTTGACTCTTTTAAAAGC
TTTAGTTCGACAACAACTTCCAGAAAAGTATAAAGAAATCTTTTTTGATCAATCAAAAAACGGATATGC
AGGTTATATTGATGGGGGAGCTAGCCAAGAAGAATTTTATAAATTTATCAAACCAATTTTAGAAAAAAT
GGATGGTACTGAGGAATTATTGGTGAAACTAAATCGTGAAGATTTGCTGCGCAAGCAACGGACCTTTG
ACAACGGCTCTATTCCCCATCAAATTCACTTGGGTGAGCTGCATGCTATTTTGAGAAGACAAGAAGAC
TTTTATCCATTTTTAAAAGACAATCGTGAGAAGATTGAAAAAATCTTGACTTTTCGAATTCCTTATTATG
TTGGTCCATTGGCGCGTGGCAATAGTCGTTTTGCATGGATGACTCGGAAGTCTGAAGAAACAATTACC
CCATGGAATTTTGAAGAAGTTGTCGATAAAGGTGCTTCAGCTCAATCATTTATTGAACGCATGACAAAC
TTTGATAAAAATCTTCCAAATGAAAAAGTACTACCAAAACATAGTTTGCTTTATGAGTATTTTACGGTTT
ATAACGAATTGACAAAGGTCAAATATGTTACTGAAGGAATGCGAAAACCAGCATTTCTTTCAGGTGAA
CAGAAGAAAGCCATTGTTGATTTACTCTTCAAAACAAATCGAAAAGTAACCGTTAAGCAATTAAAAGA
AGATTATTTCAAAAAAATAGAATGTTTTGATAGTGTTGAAATTTCAGGAGTTGAAGATAGATTTAATGCT
TCATTAGGTACCTACCATGATTTGCTAAAAATTATTAAAGATAAAGATTTTTTGGATAATGAAGAAAATG
AAGATATCTTAGAGGATATTGTTTTAACATTGACCTTATTTGAAGATAGGGAGATGATTGAGGAAAGAC
TTAAAACATATGCTCACCTCTTTGATGATAAGGTGATGAAACAGCTTAAACGTCGCCGTTATACTGGTT
GGGGACGTTTGTCTCGAAAATTGATTAATGGTATTAGGGATAAGCAATCTGGCAAAACAATATTAGATT
TTTTGAAATCAGATGGTTTTGCCAATCGCAATTTTATGCAGCTGATCCATGATGATAGTTTGACATTTAA
AGAAGACATTCAAAAAGCACAAGTGTCTGGACAAGGCGATAGTTTACATGAACATATTGCAAATTTAG
CTGGTAGCCCTGCTATTAAAAAAGGTATTTTACAGACTGTAAAAGTTGTTGATGAATTGGTCAAAGTAA
TGGGGCGGCATAAGCCAGAAAATATCGTTATTGAAATGGCACGTGAAAATCAGACAACTCAAAAGGG
CCAGAAAAATTCGCGAGAGCGTATGAAACGAATCGAAGAAGGTATCAAAGAATTAGGAAGTCAGATT
CTTAAAGAGCATCCTGTTGAAAATACTCAATTGCAAAATGAAAAGCTCTATCTCTATTATCTCCAAAAT
GGAAGAGACATGTATGTGGACCAAGAATTAGATATTAATCGTTTAAGTGATTATGATGTCGATCACATTG
TTCCACAAAGTTTCCTTAAAGACGATTCAATAGACAATAAGGTCTTAACGCGTTCTGATAAAAATCGTG
GTAAATCGGATAACGTTCCAAGTGAAGAAGTAGTCAAAAAGATGAAAAACTATTGGAGACAACTTCTA
AACGCCAAGTTAATCACTCAACGTAAGTTTGATAATTTAACGAAAGCTGAACGTGGAGGTTTGAGTGA
ACTTGATAAAGCTGGTTTTATCAAACGCCAATTGGTTGAAACTCGCCAAATCACTAAGCATGTGGCAC
AAATTTTGGATAGTCGCATGAATACTAAATACGATGAAAATGATAAACTTATTCGAGAGGTTAAAGTGA
TTACCTTAAAATCTAAATTAGTTTCTGACTTCCGAAAAGATTTCCAATTCTATAAAGTACGTGAGATTAA
CAATTACCATCATGCCCATGATGCGTATCTAAATGCCGTCGTTGGAACTGCTTTGATTAAGAAATATCCA
AAACTTGAATCGGAGTTTGTCTATGGTGATTATAAAGTTTATGATGTTCGTAAAATGATTGCTAAGTCTG
AGCAAGAAATAGGCAAAGCAACCGCAAAATATTTCTTTTACTCTAATATCATGAACTTCTTCAAAACAG
AAATTACACTTGCAAATGGAGAGATTCGCAAACGCCCTCTAATCGAAACTAATGGGGAAACTGGAGA
AATTGTCTGGGATAAAGGGCGAGATTTTGCCACAGTGCGCAAAGTATTGTCCATGCCCCAAGTCAATAT

TGTCAAGAAAACAGAAGTACAGACAGGCGGATTCTCCAAGGAGTCAATTTTACCAAAAAGAAATTCG
GACAAGCTTATTGCTCGTAAAAAAGACTGGGATCCAAAAAAATATGGTGGTTTTGATAGTCCAACGGT
AGCTTATTCAGTCCTAGTGGTTGCTAAGGTGGAAAAAGGGAAATCGAAGAAGTTAAAATCCGTTAAAG
AGTTACTAGGGATCACAATTATGGAAAGAAGTTCCTTTGAAAAAAATCCGATTGACTTTTTAGAAGCTA
AAGGATATAAGGAAGTTAAAAAAGACTTAATCATTAAACTACCTAAATATAGTCTTTTTGAGTTAGAAA
ACGGTCGTAAACGGATGCTGGCTAGTGCCGGAGAATTACAAAAAGGAAATGAGCTGGCTCTGCCAAG
CAAATATGTGAATTTTTTATATTTAGCTAGTCATTATGAAAAGTTGAAGGGTAGTCCAGAAGATAACGAA
CAAAAACAATTGTTTGTGGAGCAGCATAAGCATTATTTAGATGAGATTATTGAGCAAATCAGTGAATTT
TCTAAGCGTGTTATTTTAGCAGATGCCAATTTAGATAAAGTTCTTAGTGCATATAACAAACATAGAGACA
AACCAATACGTGAACAAGCAGAAAATATTATTCATTTATTTACGTTGACGAATCTTGGAGCTCCCGCTG
CTTTTAAATATTTTGATACAACAATTGATCGTAAACGATATACGTCTACAAAAGAAGTTTTAGATGCCAC
TCTTATCCATCAATCCATCACTGGTCTTTATGAAACACGCATTGATTTGAGTCAGCTAGGAGGTGACTG
ATGAAGCCAGTTACCTTCGGAAAAAGAGTTGGTAGCTCTTGATCCGGCAAACAAACCACCGCTGGTA
GCGGTGGTTTTTTTGTTTGCAAGCAGCAGATTACGCGCAGAAAAAAAGGATCTCAAGAAGATCCTTTG
ATCTTTTCTACGGGGTCTGACGCTCAGTGGAACGAAAACTCACGTTAAGGGATTTTGGTCATGAGATT
ATCAAAAAGGATCTTCACCTAGATCCTTTTAAATTAAAAATGAAGTTTTAAATCAATCTAAAGTATATAT
GAGTAAACTTGGTCTGACAATCGATGCAAGGGTTGGTTTGCGCATTCACAGTTCTCCGCAAGAATTGA
TTGGCTCCAATTCTTGGAGTGGTGAATCCGTTAGCGAGGTGCCGCCGGCTTCCATTCAGGTCGAGGTG
GCCCGGCTCCATGCACCGCGACGCAACGCGGGGAGGCAGACAAGGTATAGGGCGGCGCCTACAATCC
ATGCCAACCCGTTCCATGTGCTCGCCGAGGCGGCATAAATCGCCGTGACGATCAGCGGTCCAGTGATC
GAAGTTAGGCTGGTAAGAGCCGCGAGCGATCCTTGAAGCTGTCCCTGATGGTCGTCATCTACCTGCCT
GGACAGCATGGCCTGCAACGCGGGCATCCCGATGCCGCCGGAAGCGAGAAGAATCATAATGGGGAAG
GCCATCCAGCCTCGCGTCGCGAACGCCAGCAAGACGTAGCCCAGCGCGTCGGCCGCCATGCCGGCGA
TAATGGCCTGCTTCTCGCCGAAACGTTTGGTGGCGGGACCAGTGACGAAGGCTTGAGCGAGGGCGTG
CAAGATTCCGAATACCGCAAGCGACAGGCCGATCATCGTCGCGCTCCAGCGAAAGCGGTCCTCGCCG
AAAATGACCCAGAGCGCTGCCGGCACCTGTCCTACGAGTTGCATGATAAAGAAGACAGTCATAAGTG
CGGCGACGATAGTCATGCCCCGCGCCCACCGGAAGGAGCTGACTGGGTTGAAGGCTCTCAAGGGCAT
CGGTCGGCGCTCTCCCTTATGCGACTCCTGCATTAGGAAGCAGCCCAGTAGTAGGTTGAGGCCGTTGA
GCACCGCCGCCGCAAGGAATGGTGCATGTAAGGAGATGGCGCCCAACAGTCCCCCGGCCACGGGGCC
TGCCACCATACCCACGCCGAAACAAGCGCTCATGAGCCCGAAGTGGCGAGCCCGATCTTCCCCATCGG
TGATGTCGGCGATATAGGCGCCAGCAACCGCACCTGTGGCGCCGGTGATGCCGGCCACGATGCGTCCG
GCGTAGAGAATCCACAGGACGGGTGTGGTCGCCATGATCGCGTAGTCGATAGTGGCTCCAAGTAGCGA
AGCGAGCAGGACTGGGCGGCGGCCAAAGCGGTCGGACAGTGCTCCGAGAACGGGTGCGCATAGAAA
TTGCATCAACGCATATAGCGCTAGCAGCACGCCATAGTGACTGGCGATGCTGTCGGAATGGACGATATC
CCGCAAGAGGCCCGGCAGTACCGGCATAACCAAGCCTATGCCTACAGCATCCAGGGTGACGGTGCCG
AGGATGACGATGAGCGCATTGTTAGATTTCATACTCTTCCTTTTTCAATATTATTGAAGCATTTATCAGG
GTTATTGTCTCATGAGCGGATACATATTTGAATGTATTTAGAAAAATAAACAAATAGGGGTTCCGCGCAC
ATTTCCCCGAAAAGTGCCACCTG
